# Influence of Non-canonical DNA Bases on the Genomic Diversity of *Tevenvirinae*

**DOI:** 10.3389/fmicb.2021.632686

**Published:** 2021-04-06

**Authors:** Nikita A. Nikulin, Andrei A. Zimin

**Affiliations:** ^1^Laboratory of Bacteriophage Biology, G.K. Skryabin Institute of Biochemistry and Physiology of Microorganisms, Pushchino Scientific Center for Biological Research of the Russian Academy of Sciences, Pushchino, Russia; ^2^Laboratory of Molecular Microbiology, G.K. Skryabin Institute of Biochemistry and Physiology of Microorganisms, Pushchino Scientific Center for Biological Research of the Russian Academy of Sciences, Pushchino, Russia

**Keywords:** *Tevenvirinae*, phages, non-canonical bases, modified bases, genome diversity

## Abstract

The *Tevenvirinae* viruses are some of the most common viruses on Earth. Representatives of this subfamily have long been used in the molecular biology studies as model organisms – since the emergence of the discipline. *Tevenvirinae* are promising agents for phage therapy in animals and humans, since their representatives have only lytic life cycle and many of their host bacteria are pathogens. As confirmed experimentally, some *Tevenvirinae* have non-canonical DNA bases. Non-canonical bases can play an essential role in the diversification of closely related viruses. The article performs a comparative and evolutionary analysis of *Tevenvirinae* genomes and components of *Tevenvirinae* genomes. A comparative analysis of these genomes and the genes associated with the synthesis of non-canonical bases allows us to conclude that non-canonical bases have a major influence on the divergence of *Tevenvirinae* viruses within the same habitats. Supposedly, *Tevenvirinae* developed a strategy for changing HGT frequency in individual populations, which was based on the accumulation of proteins for the synthesis of non-canonical bases and proteins that used those bases as substrates. Owing to this strategy, ancestors of *Tevenvirinae* with the highest frequency of HGT acquired genes that allowed them to exist in a certain niche, and ancestors with the lowest HGT frequency preserved the most adaptive of those genes. Given the origin and characteristics of genes associated with the synthesis of non-canonical bases in *Tevenvirinae*, one can assume that other phages may have similar strategies. The article demonstrates the dependence of genomic diversity of closely related *Tevenvirinae* on non-canonical bases.

## Introduction

Bacteriophages are some of the most abundant biological objects on Earth (Wommack and Colwell, 2000). Their reproductive cycles, as well as their interactions with the hosts and the environment, are very diverse. The advancement of modern sequencing techniques allows researchers to examine individual genomes, metaviromes, and metagenomes – which, in turn, makes it possible to conduct detailed studies of evolutionary, coevolutionary, and ecological processes in bacteriophages.

One of the first routinely studied bacteriophages were T-even phages: T2, T4, T6 – which played an essential role in the development of molecular genetics (Abedon, 2000). According to ICTV Taxonomy (2019), they belong to the *Tequatrovirus* genus, *Tevenvirinae* subfamily, *Myoviridae* family, *Caudovirales* order. In addition to these T-even bacteriophages, many representatives of *Tevenvirinae* (for example, RB phage) were used as objects in the studies of biology of bacterial viruses, (Russell, 1967). These bacteriophages are considered to be in the group of the most promising candidates for phage therapy (Sarker et al., 2012). The development of sequencing technologies has shown a wide distribution of *Tevenvirinae* and related phages in nature, as well as a great variety of their host bacteria, which include animal and human pathogens (Petrov et al., 2010; Sullivan et al., 2010; Roux et al., 2015).

A wide representation of *Tevenvirinae* genomes in the online databases has made it possible to analyze their general composition and structural organization, i.e., their pan-genome (Medini et al., 2005; Petrov et al., 2010). The analysis has revealed conserved regions of the *Tevenvirinae* genome typical of the subfamily members (core-genome), as well as variable zones (quasi-core and plastic genomes) (Comeau et al., 2007; Petrov et al., 2010). Comprehensive knowledge of protein functions of some members of the subfamily (e.g., *Escherichia* virus T4) has allowed researchers to identify the corresponding protein homologs, whose genes are located in different regions of pan-genome (Petrov et al., 2010). On the basis of these data, studies of the evolutionary divergence of *Tevenvirinae* have become possible.

One of the experimentally confirmed features of *Tevenvirinae* is the presence of non-canonical (modified) DNA bases in some of their representatives (Weigele and Raleigh, 2016; Thomas et al., 2018; Hutinet et al., 2019). There are also data on the putative proteins that are highly likely to participate in the biosynthesis of non-canonical bases in *Tevenvirinae* – however, the functions of these proteins have yet to be experimentally confirmed (Iyer et al., 2013; Hutinet et al., 2019). Some of the non-canonical bases are supposed to serve as a barrier to gene transfer and, therefore, to be a speciation factor among related *Tevenvirinae* phages (Petrov et al., 2010; Thomas et al., 2018). As shown earlier, transfer of genetic information between phages with different sets of DNA bases can be very difficult (Russell, 1967; Thomas et al., 2018).

To better understand the evolutionary divergence of *Tevenvirinae*, as well as the influence of non-canonical DNA base systems on this process, we have conducted a comparative genome-wide and pan-genomic study of *Tevenvirinae* viruses registered in the NCBI database. In the study, we compare data of phylogenetic, phylogenomic, and bipartite network analyses to identify evolutionary changes in the processes of genetic information exchange. In addition, we have performed a comparative analysis of the genetic loci involved in the synthesis of non-canonical bases and have searched for their homologs using BLAST (Madden et al., 1996) – to identify the specifics of inheritance of such genes.

Altogether, the analysis conducted has allowed us to identify specific genetic and genomic changes in the systems of *Tevenvirinae* responsible for the composition of their sets of DNA bases. This contributes to our understanding of the evolution and divergence of this subfamily of phages and may help to identify similar systems in the phages of other phylogenetic groups.

## Materials and Methods

### Phage Genome Sequences

Two hundred and five complete genomes available in the NCBI database as of July 29, 2019 have been uploaded in the “gbk” format. The genomes met the parameters of (complete genome) and [Taxonomy ID: 1198136 (Organism “*Tevenvirinae*”)] and were used for further analysis. The GenBank accession numbers, phage names, bacteria used for phage isolation, sources of isolation, and available links are given in Supplementary Table 1 of the Supplementary Section.

### Genome Analysis

#### Genome Clustering and Annotation

The annotations of 205 *Tevenvirinae* genomes deposited in GenBank were verified according to the following procedure. The annotations were checked for the presence of predicted ORFs and, if they were absent, the genomes were annotated automatically using the Genome Annotation PATRIC service with the parameters (Domain: Viruses) and (Annotation recipe: Phage) (Brettin et al., 2015; Wattam et al., 2018). The homologs of the translated genome frames were clustered using the GET_HOMOLOGUES (get_homologues.pl) script with the parameters of [minimal coverage (-C): 40%], [minimal identity (-S): 50%], [minimal *e*-value (-E): standard (1e-05)], and [algorithm clustering: COGtriangle] (Kristensen et al., 2010; Contreras-Moreira and Vinuesa, 2013). The minimal values of the coverage and identity parameters were selected on the basis of the best clustering of translated ORFs of core genome of T4-related bacteriophages determined by Petrov et al., 2010. If translated ORFs of core genome were absent in the analyzed genome, its sequence was automatically reannotated using PATRIC and then verified again. If ORF of a core gene was shifted, disrupted or absent, the genome was excluded from further analysis.

The functions of the proteins of core proteome were predicted on the basis of clustering of the corresponding homologs with the translated ORFs of the genome of *Escherichia* virus T4 (NC_000866.4) (Miller et al., 2003).

#### Pan-Genomic Analysis

To analyze the homolog proteins of Tevenvirinae proteome obtained by the get_homologues.pl script, we used a pan-genomic matrix of presence/absence of clustered sequences, which was generated using the compare_clusters.pl script. The composition of the pan-genome: core-genome (Medini et al., 2005), soft-core-genome (Kaas et al., 2012), shell (Wolf et al., 2012; Vernikos et al., 2015), cloud (Wolf et al., 2012) – was determined using the parse_pangenome_matrix.pl script. A heat map of pan-genome clusters was built using the plot_matrix_heatmap.sh script.

### Phylogenomic Tree, Phylogenetic Tree, Pan-Genomic Tree and Bipartite Network

On the basis of the binary matrix of cluster presence/absence, a pan-genomic tree was constructed using the estimate_pangenome_phylogenies.sh GET_PHYLOMARKERS script (Vinuesa et al., 2018) with 100 replications (-r). A phylogenetic tree was constructed from MUSCLE-aligned sequences of the main head protein, which is used as a phylogenetic marker of this group of viruses (Comeau and Krisch, 2008), with the help of W-IQ-TREE (Trifinopoulos et al., 2016). The phylogenomic tree was constructed using VICTOR (Meier-Kolthoff and Göker, 2017) and 100 nucleotide sequences of genomes (tool limitation), which formed separate clades along the pan-genome tree. A cluster-genome bipartite network, which was based on the proteins whose genes were included in the pan-genome shell, was visualized in Cytoscape (Shannon et al., 2003).

### Cluster Analysis

The search for the homologs of clusters specific for a certain clade of genomes obtained on the pan-genomic tree and clusters presumably associated with non-canonical bases was carried out using PSI-BLAST (Altschul et al., 1997; Schäffer et al., 2001) and HMMER (Zimmermann et al., 2018).

For the initial search for homologs in PSI-BLAST, we used an amino acid sequence, belonging to one of the viral genomes, from a cluster. Then, a list of homologous proteins was obtained. Sequences of viruses, belonging to Taxonomy ID 1198136 (*Tevenvirinae*), were selected. The search was repeated (1 iteration). If the cluster was formed by a single protein and after the initial search no other homologous *Tevenvirinae* sequences were found, then the search was not repeated. The search parameters were as follows: (Max target sequences: 1000), (Word size: 2), [Matrix: BLOSUM45 (in case of CPU exceeding, BLOSUM62)]. To refine the annotations of clusters, in which homologs were found only among hypothetical proteins, HMMER was additionally used. A search was performed with the standard parameters of the nr30 database and a matrix based on the protein sequences aligned with MUSCLE (Edgar, 2004), which is included in the UGENE toolkit (Okonechnikov et al., 2012).

Individual regions of the genomes, containing genes associated with the synthesis of non-canonical bases, were compared using Easyfig (Sullivan et al., 2011).

### Additional Genome Analysis

To further verify the results, a search for *Tevenvirinae* genomes among uncultured viruses from GenBank and IMG/VR (Roux et al., 2021) databases was conducted.

For GenBank, PSI-BLAST was used with the parameters: (Max target sequences: 1000), (Word size: 2), (Matrix: BLOSUM45), [Organism: Viruses (taxid:10239)]. A PSSM matrix was created based on the amino-acid sequences of the *Tevenvirinae* (taxid:1198136) proteins that comprise the core. The genomes of the uncultured viruses, whose proteins were found on the first PSI-BLAST iteration, were checked for the genome completeness: only those genomes which were marked as (complete genome) were selected for further analysis.

For IMG/VR, Viral BLAST was used. The search parameters were as follows: (Program: blastn), (Blast Database: Viral Sequence blast database), (*E*-value: 1e-2). The twenty genomes of the *Tevenvirinae* viruses, belonging to groups determined by pan-genomic analysis, were used as query sequences. Of the genomes found, only those that met the criterion for high quality genome (Quality: High-quality) were selected for further analysis.

We have also uploaded and included into the dataset the *Tevenvirinae* genomes deposited within the GenBank database from July 29, 2019 to January 30, 2021.

Clustering and selection of genomes were carried out as described in Genome Clustering and Annotation. Clusters determined by the results of pan-genomic analysis were used as the core genome. Phylogenomic and pan-genomic trees were built as described in the section “Phylogenomic Tree, Phylogenetic Tree, Pan-genomic Tree and Bipartite Network.”

## Results

### Clustering and Selection of Genomes

A survey of the annotations of genomes deposited in GenBank showed that Escherichia phages CF2 and HP3 lacked predicted ORFs, so they were reannotated before clustering. In total, the procedure of clustering was carried out three times: (1) clustering of initially annotated genomes (with the exception of CF2 and HP3 genomes); (2) clustering of reannotated genomes that initially lacked core genome components; and (3) clustering of selected genomes. The numbers of pan-genome components in each of the genomes, which were obtained by using the parse_pangenome_matrix.pl script, and the heat map of the cluster presence/absence are given in Supplementary Data Sheet 1 and Supplementary Figure 1.

Clustering of initially annotated genomes (1) showed that 79 of them lacked some core-genome genes. After clustering with reannotated genomes (2), we found that the core protein of the late transcription co-activator (gp33 in the T4 phage) of Vibrio phages, belonging to the genus *Schizotequatrovirus*, did not form a common homologous cluster with the gp33 co-activator proteins of other *Tevenvirinae*. Since these clusters of proteins had very low homology with each other, the homologs of the late transcription co-activator of Vibrio phages as a core-gene were not taken into account when genomes were selected. A similar situation was with the genes of tail fibril proteins (gp35, gp36, and gp37). These genes have high mosaicism and are often clustered with non-homologous genes of fibrils of other phages (Petrov et al., 2010).

At the second stage, the genomes Aeromonas phage phiAS5, Shigella phage SHFML-26, Shigella phage Sf25, Vibrio phage nt-1, and Vibrio phage VH7D were not used for the analysis. Due to the shift of the reading frames in a number of genes, they lacked the necessary components of the core genome.

In addition, when examining the soft core of the pan genome, we found that in the genome of Aeromonas phage Ah1 there was a shift in the reading frame in the gene for the head assembly protein (gp40 in phage T4); therefore, this phage was not used for the analysis either.

We clustered the remaining 199 genomes again. As a result, they all contained the necessary set of core genes (with the exception of the genes described above).

At (3) stage 57499 amino acid sequences were used, 4530 homologous clusters were obtained: 61 clusters were included in the core, 76 (taking into account the core-genome clusters) in the soft core, 1966 in the shell, and 2488 in the cloud. As a result, 79 genomes were reannotated, 7 of them were removed and 199 genomes were used for further analysis.

### Phylogenetic Tree, Phylogenomic Tree, Pan-Genomic Tree and Bipartite Network

**FIGURE 1:**
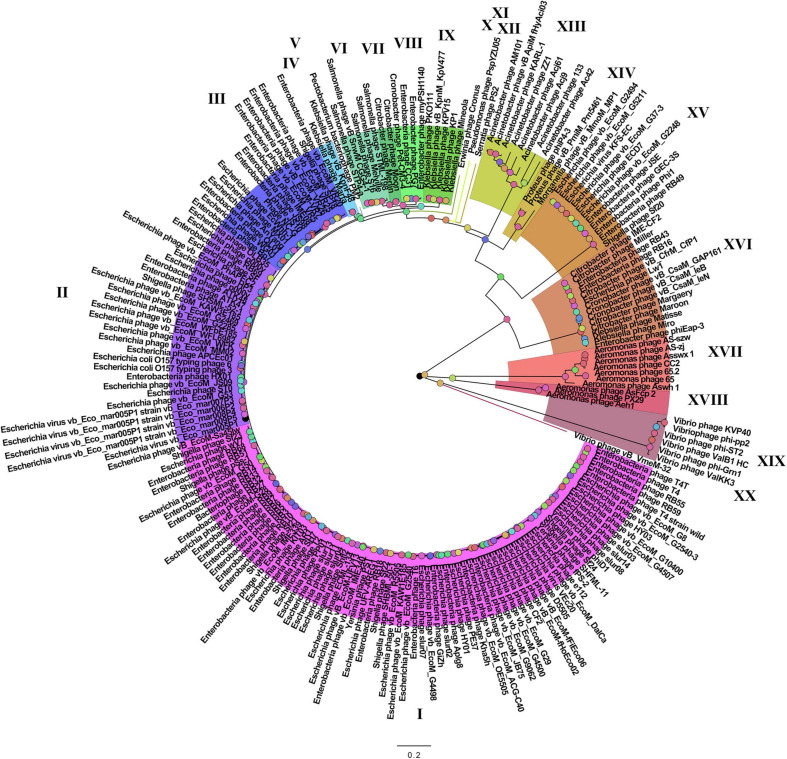
The pan-genomic tree of *Tevenvirinae*. Selective clades are marked with Roman numerals and colors. The statistical values presented along with the tree in Supplementary Figure 2 are marked with colored circles.

**FIGURE 2:**
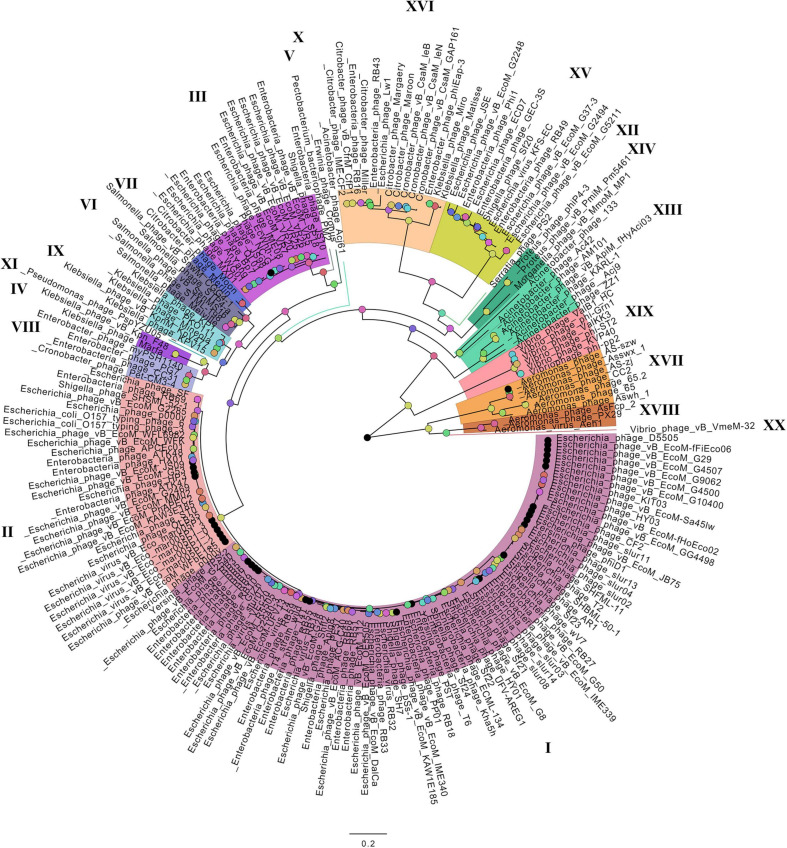
The phylogenetic tree of the main head protein of *Tevenvirinae*. Selective clades are marked with Roman numerals and colors. The statistical values presented along with the tree in Supplementary Figure 3 are marked with colored circles.

**FIGURE 3:**
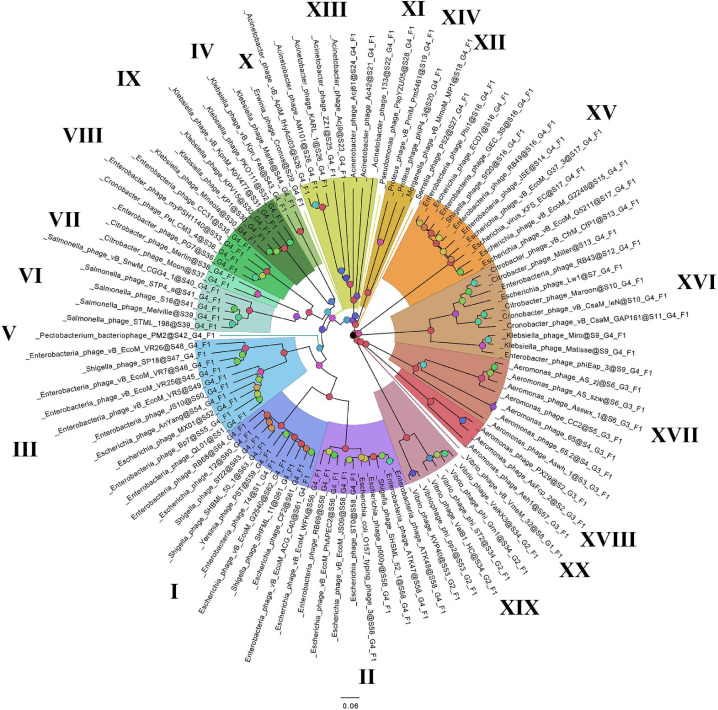
The phylogenomic tree of *Tevenvirinae*. Selective clades are marked with Roman numerals and colors. The statistical values presented along with the tree in Supplementary Figure 4 are marked with colored circles.

On the basis of the binary matrix of cluster presence/absence, a pan-genomic tree was built (Figure 1). The best model: GTR2 + FO + I + G4. Log-likelihood of the consensus tree: −41953.89217. Figure 2 shows a phylogenetic tree constructed on the basis of homologous sequences of the main head protein. The best model: LG + F + I + G4. The best score found: −15512.735. On the basis of the genome nucleotide sequences, a phylogenomic tree was built (Figure 3). As verified by statistical analysis, the main clades on all trees were identified with high probabilities. Exceptions are some minor clades on the pan-genomic tree, which are represented by a single genome: Pseudomonas phage PspYZU05 [aBayes support (Anisimova et al., 2011) 0.333; ultrafast bootstrap support (Hoang et al., 2018) 15%], Serratia phage PS2 (0.379; 31%, respectively). Groups of viruses which form separate clades are marked by different colors and also by Roman figures (descriptions of groups are given below and in Supplementary Data Sheet 4). Colored circles denote statistical values given in Supplementary Figures 2–4.

**FIGURE 4:**
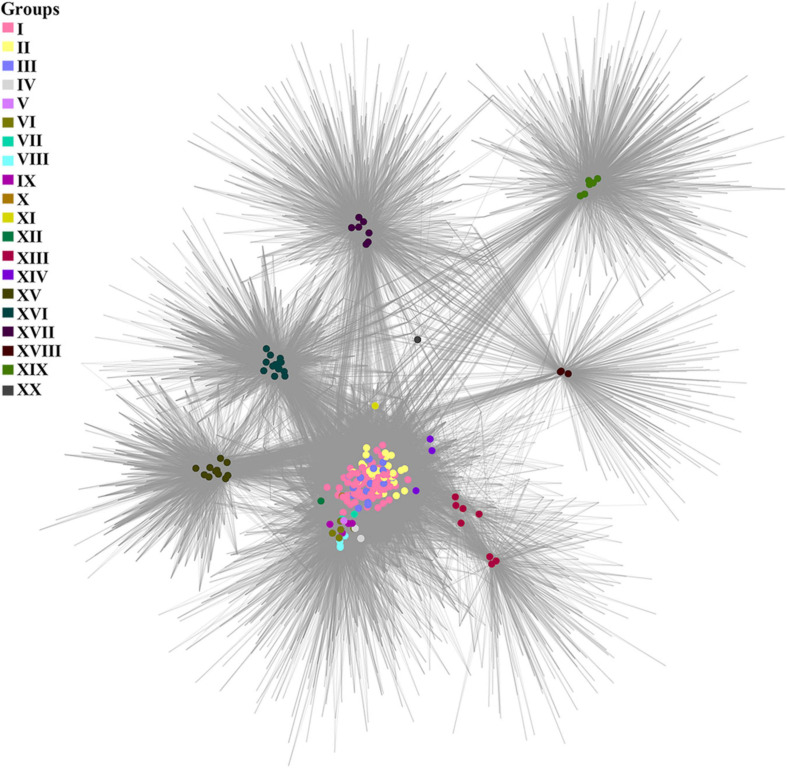
A cluster-genome bipartite network of the pan-genome envelope of *Tevenvirinae*. Genomes that belong to the same cluster (group) are marked with colored circles. The connected nodes represent individual clusters of translated ORF homologs.

A cluster-genome bipartite network based on the clusters of the pan-genome envelope is shown in Figure 4. Colored nodes are genomes of viruses, color is the group to which the virus belongs. Homologous clusters are represented by the intersections of edges.

Analyzing the pan-genomic tree, we identified 20 *Tevenvirinae* groups. The following criteria were used to divide genomes into separate groups:

(1) Each group forms a separate clade on the pan-genome tree.
(2) Each group has GSCs (homologous clusters with nucleotide sequences present in all genomes of the group and absent in the genomes of other groups).
(3) NCBI phage taxonomic classification, which can be accessed in the Taxonomy Database, should also be taken into account. Phages, belonging to one genus and forming a separate clade with clearly identifiable GSC, were referred to one group. The only exception is Group 3, which contains members of two phage genera, *Dhakavirus* and *Gaprivervirus*. This is because the clade formed by the members of the *Dhakavirus* genus does not have identifiable GSC.

In the case of phages that have not yet been classified down to the genus level, only the first two criteria were applied.

It should be noted that – as shown by the analysis of the tree and Supplementary Table 1 – phages from the groups represented by more than one genome were isolated from related (at the level of order, family, or genus) host bacteria: *Enterobacteriales* (I–III, VIII, XV, XVI), *Morganellaceae* (XIV), *Klebsiella* (IV, IX), *Salmonella* (VI), *Citrobacter* (VII), *Acinetobacter* (XIII), *Aeromonas* (XVII, XVIII), *Vibrio* (XIX).

A comparative analysis of the pan-genomic, phylogenomic, and phylogenetic trees showed that in all cases, the representatives of the identified groups were in the same clades – with the exception of group XIII, a representative of which (Acinetobacter phage Acj61) formed a separate clade on the phylogenetic tree. The main differences between the three trees were in the clade hierarchy and hierarchy of representatives within the clades. These differences may be due to the fact that pan-genomic and phylogenomic trees reflect the exchange of genetic information between *Tevenvirinae* phages, whereas phylogenetic tree shows the evolution of a single conservative protein, the main protein of the head. Overall, these trees agree with the views on *Tevenvirinae* bacteriophages presented in other publications (Tétart et al., 2001; Ignacio-Espinoza and Sullivan, 2012).

As noted by Gautreau et al., 2020, the pan-genome shell is of greatest interest in examining the dynamics of the development of bacterial genomes, reflecting their interactions with the ecological niche. This statement may be even more relevant toward bacteriophages, since their evolution is modular (Iranzo et al., 2016). To analyze the envelope, we constructed a bipartite network based on the clusters of this component of pan-genome. One can see that, in general, the cluster network coincides with the pan-genomic tree, yet there is a number of differences. For example, groups I–III of the network are “intermixed” with each other, which is not surprising given the specifics of the ecological conditions of their habitats. In this respect, it is worth to note the genome of Vibrio phage vB_VmeM-32, the only representative of the XX group. It has the smallest number of common clusters with other genomes. However, the majority of these clusters are characteristic of the genomes of most other groups, as indicated by the location of the phage in the network and its connections.

To get more information about the phage groups identified, we analyzed the clusters of homologs specific to each group.

To characterize these clusters (GSCs, group-specific clusters), we searched for homologous sequences. The search revealed (1) GSCs that did not have homologs among the proteins registered in the Non-Redundant Protein Sequences (nr) database; (2) GSCs that had homologs among hypothetical proteins of other organisms; and (3) GSCs that had homologs among proteins of other organisms with a predicted function. The numbers of clusters identified according to these criteria are given in Table 1. Characteristics of clusters of each group are presented in Supplementary Text 1 and Supplementary Table 2.

**TABLE 1:** The number of group-specific clusters.

**Group**	**Total number of GSCs**	**Number of GSCs with homologs of hypothetical proteins**	**Number of GSCs with homologs of proteins whose functions are known**
I	2	1	1
II	4	2	0
III	1	0	0
IV	27	7	1
V	43	14	2
VI	12	1	0
VII	22	5	0
VIII	28	23	1
IX	23	23	0
X	134	7	3
XI	75	8	6
XII	128	15	4
XIII	7	2	1
XIV	20	14	4
XV	99	25	5
XVI	69	13	4
XVII	104	27	4
XVIII	146	10	8
XIX	219	39	32
XX	137	31	19

### Non-canonical Bases and Related Proteins

As mentioned above, bacteriophages of group XIX have GSCs associated with the synthesis of modifications of 7-deazaguanine. As confirmed experimentally, some *Tevenvirinae* phages can have other non-canonical bases: glucosylated ^hm^dC (T-even bacteriophages from group I); arabinosylated ^hm^dC (RB69 from group II). Correspondingly, we have searched *Tevenvirinae* for the protein homologs that are involved – either *in vitro* or *in silico*, according to the literature data (Petrov et al., 2010; Iyer et al., 2013; Thomas et al., 2018; Hutinet et al., 2019) – in the synthesis of such bases (or are associated with them in any other way). A list of such proteins and their genes is presented in Table 2.

**TABLE 2:** Proteins associated or hypothetically associated with non-canonical bases.

**Gene/Locus (phage)**	**Protein/Putative protein (domain)**	**Function/Possible function**	**Non-canonical base**
42 (Enterobacteria phage T4)	HMC-transferase (Lamm et al., 1987)	^hm^dCMP synthesis	Modifications of ^hm^C
α-gt (Enterobacteria phage T4)	α-glucosyl transferase (Gold and Schweiger, 1969)	α-glucosylation of ^hm^dCMP in DNA	
β -gt (Enterobacteria phage T4)	β-glucosyl transferase (Gold and Schweiger, 1969)	β-glucosylation of ^hm^dCMP in DNA	
βα-gt (Enterobacteria phage T2)	β-glucosyl-1,6-α- glucosyl transferase (Petrov et al., 2010)	β-glucosylation of α-glucosylated ^hm^dCMP in DNA	
RB69p003c (Enterobacteria phage RB69)	putative arabinosyl transferase (Thomas et al., 2018)	arabinosylation of ^hm^dCMP in DNA	
Acj61p076 (Acinetobacter phage Acj61)	putative transferase (Thomas et al., 2018)	DNA hypermodification	
RB69p052 (Enterobacteria phage RB69)	homologs among Rmla and aminoglycoside 3′-phosphotransferase	probably, participates in the synthesis of UDP-arabinose (Thomas et al., 2018)	
RB69p055 (Enterobacteria phage RB69)	homologs among arabinosyl-5-phosphate isomerases	probably, participates in the synthesis of UDP-arabinose (Thomas et al., 2018)	
56 (Enterobacteria phage T4)	dCTPase – dUTPase (Kutter et al., 1975)	Increases the pool of dCMP, decreases the pool of dCTP; provides dUMP for the synthesis of dTMP	
denA (Enterobacteria phage T4)	Endonuclease II (Kutter et al., 1975, 1994)	Restricted cleavage of unmodified (dCMP-containing) DNA	
denB (Enterobacteria phage T4)	Endonuclease IV (Kutter et al., 1975, 1994)	Unrestricted cleavage of unmodified (dCMP-containing) DNA	
*alc* (Enterobacteria phage T4)	Alc protein (Drivdahl and Kutter, 1990)	Blocks transcription of unmodified (dCMP-containing) DNA	
*arn* (Enterobacteria phage T4)	Arn protein (Kim et al., 1997)	Suppress restriction of the host RM-system McR (Rgl)	
*ipI* (Enterobacteria phage T4)	internal protein I (Bair et al., 2007)	Inhibits the activity of the type-IV endonuclease RE CT596	
RB69p045 (Enterobacteria phage RB69)	Homologs among thymidylate kinases	Presumably, participate in the synthesis of arabinosyl-^ hm^dCMP	
RB69p047 (Enterobacteria phage RB69)	n/a		
RB69p049 (Enterobacteria phage RB69)	n/a		
RB69p050 (Enterobacteria phage RB69)	Homologs among peptidases		
RB69p051 (Enterobacteria phage RB69)	Presence of phosphatase domain of polynucleotide kinase		
PX29p085 (Aeromonas phage PX29)	GNAT family N-acetyltransferase (Iyer et al., 2013)	Presumably, modifies DNA	Unknown base
Aeh1ORF087c (Aeromonas phage Aeh1)	glucosyl transferase (Iyer et al., 2013)	Presumably, modifies DNA	Unknown base
KVP40.0120 (Vibrio phage KVP40)	QueD protein (Hutinet et al., 2019)	Necessary for the synthesis of 7-cyano-7-deazaguanine (PreQ0) (Hutinet et al., 2019)	Modifications of 7-deazaguanine
*folE* (Vibrio phage KVP40)	FolE protein (Hutinet et al., 2019)		
KVP40.0124 (Vibrio phage KVP40)	QueC protein (Hutinet et al., 2019)		
KVP40.0284 (Vibrio phage KVP40)	QueE protein (Hutinet et al., 2019)		
KVP40.0122 (Vibrio phage KVP40)	DpdA2 protein (Hutinet et al., 2019)	Necessary for the insertion of PreQ0 and/or PreQ1 into DNA (Hutinet et al., 2019)	
KVP40.0123 (Vibrio phage KVP40)	QueF or QueF-L protein (Hutinet et al., 2019)	Synthesis of deoxyarchaeosine or 2′-deoxy-7- aminomethyl-7-deazaguanine (Hutinet et al., 2019)	

**FIGURE 5:**
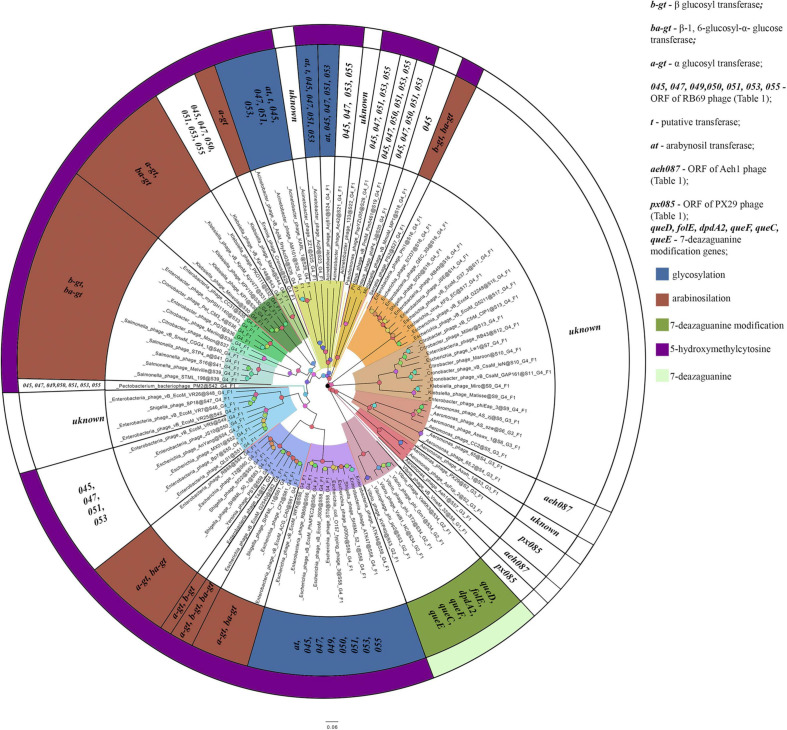
A number of gene sets associated with the synthesis of non-canonical bases. The colors indicate known modifications of non-canonical bases and the non-canonical bases as they are. The genes whose products involved or presumably involved in base modification are additionally marked.

The homologs of these proteins, belonging to the phages considered, are given in Supplementary Table 3. The overlap of group gene sets associated with the synthesis of non-canonical bases is shown in Figure 5.

**FIGURE 6:**
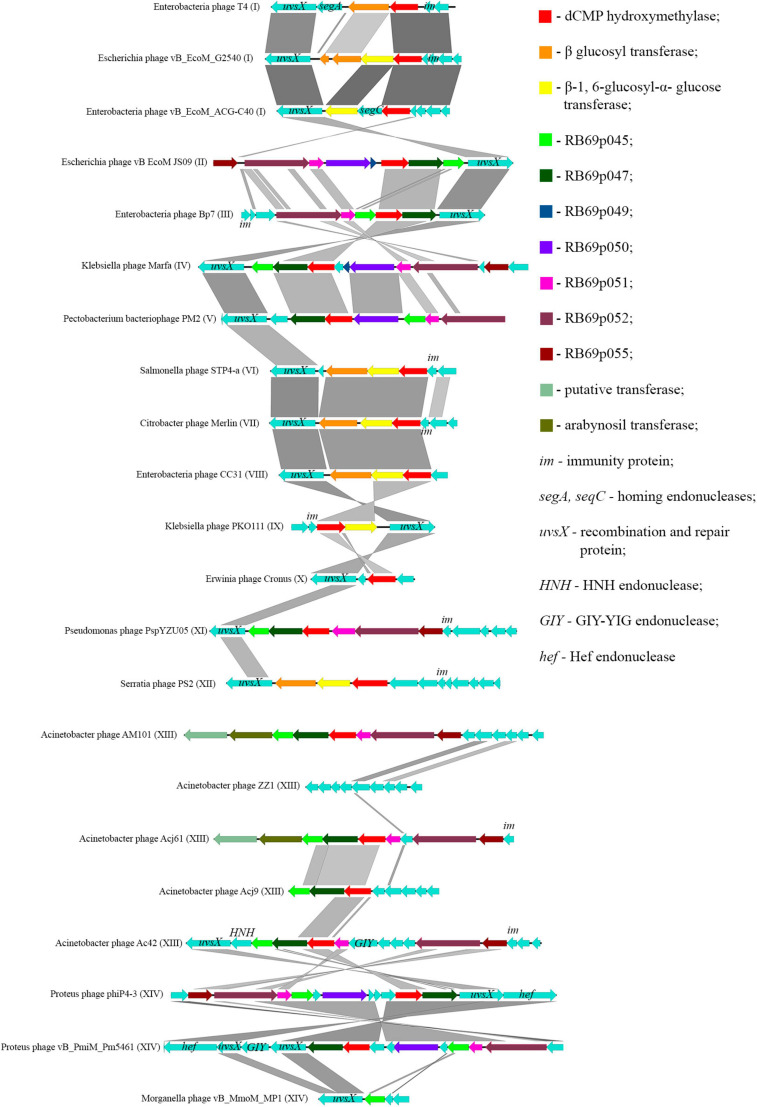
Variations in the region between the genes homologous to the genes of DNA polymerase and head vertex assembly chaperone in closely related *Tevenvirinae* that have homologs of ^hm^dC-associated proteins. The genes whose products are described in Table 2 are indicated by color; the genes whose products have homologs with known functions are additionally marked.

Thomas et al., 2018 showed that most of the genes, whose products were involved or presumably involved in arabinosylation of ^hm^dC in DNA, were located in the region between the genes encoding DNA polymerase and UvsX. The analysis of phages, having proteins associated with the synthesis of ^hm^dC and its modifications, showed that the arrangement of genes encoding those proteins was quite similar. Since the region is surrounded by the genes of head vertex assembly chaperone and DNA polymerase, we have analyzed the genome fragments between them. Figure 6 shows variations of this region revealed in closely related *Tevenvirinae* phages.

It should be noted that in Acinetobacter phage Acj9, the genes whose products are supposedly associated with the synthesis of non-canonical bases are located in a wider region of the genome: between the genes of helicase proteins and DNA polymerase (a part of the core genome). The rest of the phages had those genes located in the region considered above.

Interestingly, in the phages closely related to RB69, the gene of arabinosyl transferase is located outside the region considered. However, Acinetobacter phage AM101 and Acinetobacter phage Acj61 have two homologs of the enzyme, and their genes are located within this region.

In the majority of closely related phages, variations in the region are about presence or absence of the genes that presumably encode homing endonucleases. It was previously suggested that homing endonucleases were involved in the horizontal transfer of *Tevenvirinae* genes (Petrov et al., 2010). Therefore, one could assume that the genes associated with base modification were also involved in this transfer.

It should also be noted that the region conserved for closely related *Tevenvirinae*.

In this article, we only analyzed the region associated with ^hm^C, since the region associated with the synthesis of 7-deazaguanine modifications was considered in detail in other works (Hutinet et al., 2019). As for the phages with other possible base modifications, only one or two genes are known.

### Additional Analysis

The comparison of the results of GSC and genomic analyses revealed a clear correlation between the presence of proteins associated with non-canonical bases and phylogeny of phages, their position on the pan-genomic tree. After searching of the genomes of uncultured viruses, belonging to *Tevenvirinae*, only two genomes were selected. Most likely, this was due to the relatively large size of the *Tevenvirinae* genomes, that makes it difficult to assemble a complete genome. Also, 161 genomes were selected from *Tevenvirinae* in GenBank. A phage list and accession numbers/Scaffold IDs of these phages are given in Supplementary Table 4. The pan-genome tree and phylogenomic trees with the original and new sets of genomes are given in Supplementary Figures 5,6. As a result, the new set of genomes with the exception of the genome of the Cronobacter phage S13 formed the same groups as the original set did. Cronobacter phage S13 formed a separate clade on the trees and contained 130 unique proteins. The number of GSCs for some groups reduced, most likely due to the appearance of more evolutionary distant viruses in the clades. The total number of GSCs and a list of GSCs with homologs of each group (except for the Cronobacter S13 phage) are given in Supplementary Data Sheet 3. Clusters of known proteins associated with non-canonical bases are given in Supplementary Data Sheet 3 According to the results of additional analysis, the new genome samples contain genes associated with non-canonical bases characteristic of their group.

## Discussion

### Comparative Analysis of Genomes

On the basis of presence/absence of common protein homologs, we identified twenty groups of *Tevenvirinae* phages. The analysis of data available for the representatives of these groups showed that they were isolated from bacteria inhabiting similar ecological niches. Also, these bacteria belonged to the *Gammaproteobacteria*. Analysis of genomes in larger number also showed the conservation of the considered groups and the presence of similar characteristics in new genomes.

To consider the exchange of genetic information within *Tevenvirinae* in more detail, we analyzed connections between the clusters that form the pan-genome cloud, as well as group-specific clusters.

The analysis showed that the cluster-genome bipartite network constructed was, for the most part, similar to the pan-genomic and phylogenomic trees, but there were some differences. The biggest difference was the location of individual representatives of groups I–XIV, which did not have as many connections to the viruses of their own group as they did to the viruses of other groups. Most likely, this can be explained by the specifics of their adaptation to the environment, which is dependent on the genes of the pan-genome cloud and their products. Groups XV and XVI, however, were distant from the groups whose representatives inhabited similar ecological niches. Groups XVII and XVIII, whose phages infect Aeromonas and were isolated from similar sources, were also far apart from each other. Taking into account the characteristics of the pan-genome shell, one can assume that the common ancestors of viruses from groups XVII and XVIII possessed (or acquired in the course of the evolutionary process) an internal (intragenomic) barrier, which blocked active exchange of genes between the phages of the same ecological niche.

To characterize genomes of the identified *Tevenvirinae* groups, we searched for homologs of their group-specific clusters among the organisms whose amino acid sequences were deposited in the Non-Redundant Sequences database. As it turned out, most GSCs had no homologs among other organisms, with the exception of groups I, II, VIII, and XIV. Leaving aside the groups represented by a single phage (V, X, XI, XII, XX), the largest numbers of GSCs were discovered in the phages of groups XV–XIX. Large numbers of GSCs in the groups represented by a single phage is explained by the fact that they included clusters unique for that single genome representing the group. The survey of the organisms whose proteins had sequences similar to GSCs showed that most of them had similar ecological niches with the phages of one or another group. This may indicate that the ancestors of these organisms shared a common pool of genes.

Thus, comparison of the results of pan-genomic tree, phylogenomic tree, GSC and bipartite network analyses shows that the evolutionary divergence of *Tevenvirinae* genomes is mostly determined by the characteristics of their ecological niches.

The influence of ecological niches on the evolutionary divergence of viruses was demonstrated earlier for T4-related phages (distinguished in the literature as a superfamily), Far-T4-related phages and other bacterial viruses – on the basis of data obtained from metagenomes, metaviromes and phage genomes from various sources (Desplats and Krisch, 2003; Filée et al., 2005; Bellas and Anesio, 2013; Roux et al., 2015; Braga et al., 2020). However, for some phages, the isolation-related features of their ecological niches are not the only factors that can drive evolutionary divergence in the phage groups.

Analyzing GSCs of group XIV, we found protein homologs associated with the synthesis of modifications of 7-deazaguanine. Taking into account this finding, as well as the presence of other non-canonical bases in *Tevenvirinae* phages, we hypothesized that the emergence of different bases and their modifications in certain groups of *Tevenvirinae* ancestors was one of the factors that drive the divergence of genomes in this bacteriophage subfamily. To confirm this, we searched these viruses for the proteins that are associated with non-canonical bases.

### Influence of Non-canonical Bases on the Evolution of *Tevenvirinae*

The search for the homologs of proteins associated with non-canonical bases (Table 2) revealed that they were present in all the representatives of *Tevenvirinae*. However, the phages of groups XV and XVI, as well as some representatives of groups III (*Gaprivervirus*), XIII (Acinetobacter phage ZZ1, Acinetobacter phage 133), and XIV (Morganella phage vB_MmoM_MP1) lacked any known enzymes involved in the synthesis of such bases.

The phages of groups I–XIV (with the exception of the representatives mentioned above and Morganella phage vB_MmoM_MP1) contain homologs of the gene encoding thymidylate synthase, which is involved in the synthesis of ^hm^dCMP. The phages of groups I, VI, VII, VIII, IX, XII additionally contain genes whose products participate in ^hm^dCMP glycosylation, although the composition of these genes varies. Most of the phages of group I have α-glucosyl transferase and β-glucosyl-1,6-α-glucose transferase (bacteriophage T4 and five closely related phages have β-glucosyl transferase instead of β-glucosyl-1,6-α-glucose transferase, and Escherichia phage vB_EcoM_G2540 has genes for all three proteins). The phages of group IX also contain genes encoding α-glucosyl transferase and β-glucosyl-1,6-α-glucose transferase – like most of the phages of group I. The phages of groups VI, VII, VIII, XII have homologs of these two genes. The function of the second protein in these viruses is still unclear, since the enzyme cannot additionally modify bases by any known mechanism in the absence of α-glucosyl ^hm^dC in DNA. Therefore, either this protein modifies DNA in a different way, or its gene remains in the genomes as an atavism.

The phages of group II and XIII (with the exception of Acinetobacter phage ZZ1, Acinetobacter phage 133, and Acinetobacter phage Ac42) have homologs of proteins that are associated with arabinosylation of ^hm^dC in DNA. The phages of groups IV, V, XI and, partly, group XIV (Proteus phage phiP4-3, Proteus phage vB_PmiM_Pm5461) contain proteins that are presumably associated with this process, yet they lack any known arabinosyl transferases. Among the proteins mentioned, two are presumably involved in the synthesis of UDP-arabinose (Thomas et al., 2018) and one is a putative thymidylate kinase.

The bacteriophages of groups XVII (with the exception of Aeromonas phage AS-szw, Aeromonas phage AS-zj and Aeromonas phage Asswx 1), XVIII and XX have homologs of two genes whose products are supposedly associated with base modification. A homolog of one of the genes was common for the phages of group XX and some phages of group XVII (Aeromonas phage AsFcp 2 and Aeromonas phage PX29); and homolog of the second gene was characteristic of the members of group XVIII and some representatives of group XVIII (Aeromonas phage 65.2, Aeromonas phage 65, and Aeromonas phage Aeh1).

As mentioned above, the phages of group XIX have homologs of proteins associated with the synthesis of modifications of 7-diazaguanine. According to Hutinet et al., 2019, Vibrio phage nt-1 has a small number of these non-canonical bases substituting for guanine (in Vibrio phage nt-1 phage, guanine is only partially and rarely replaced by this modifications). No such data are available for the remaining representatives of group XIX. Since they differ from Vibrio phage nt-1 in terms of the genes whose products are involved in the synthesis of 7-diazaguanine modifications, the percentage of substitutions may differ as well.

Some *Tevenvirinae* phages lack proteins associated with the synthesis of non-canonical bases, yet they have homologs of the genes whose products are dependent on these bases. Namely, these are homologs of DenA, DenB (Kutter et al., 1975, 1994), Alc (Drivdahl and Kutter, 1990), and dCTPase – dUTPase (Kutter et al., 1975). Their functions are listed in Table 2. It should be noted that the presence of DenA and dCTPase – dUTPase homologs is not essential for the synthesis and transfer of DNA with a canonical-nucleotide composition. As shown experimentally, representatives of groups XV and XVI (namely, phages RB43 and RB49) are able to carry out general transduction in the bacteria that contain canonical DNA (Taniashin et al., 2003). At the same time, these phages have genes encoding DenA and dCTPase – dUTPase homologs. The activity of the protein Alc can be inhibited, as shown by Kashlev et al., 1993, by the methylation of CpG sequences in the vicinity of its locus. As for DenB, we did not find any examples that would demonstrate inhibition of its endonuclease activity. Moreover, DenB was shown to play a significant role in the hydrolysis of DNA with a canonical base composition (Kutter et al., 1975, 1994; Petrov et al., 2006). Thus, the presence of genes of DenB and Alc homologs may indicate the presence of non-canonical bases in the viral DNA, whereas the presence of DenA and dCTP – dUTPase homologs only partially indicates this – especially in viruses that are most distant from the phages of group I.

The representatives of group III, forming the genus *Gaprivervirus*, were found to have homologs of all the four proteins described above. It is possible that these phages have an unknown protein associated with the synthesis of non-canonical bases. The same assumption can be made in respect to Acinetobacter phage ZZ1 and Acinetobacter phage 133 (which have homologs of DenB, Alc, and dCTPase – dUTPase) and for Aeromonas phages AS-szw, AS-zj, and Asswx 1 (these phages have homologs of denA and Alc). The phages of groups XV and XVI, as shown earlier, only have DenA and dCTP – dUTPase homologs. Phages of these two groups do not have any known proteins associated with the synthesis of non-canonical bases – yet the analysis of their GSCs revealed homologs of C-5 cytosine-specific DNA methylase (in the phages of group XV) and DNA-cytosine methyltransferase (in the phages of group XVI). These enzymes can potentially inhibit the activity of DenA and dCTP – dUTPase enzymes. Further investigation of these phages can clarify how they protect themselves from the action of enzymes associated with non-canonical bases (whose genes they carry in their genomes).

A comparative analysis of the sets of proteins associated with non-canonical bases and the relationships between *Tevenvirinae* phages showed that closely related phages had enzymes involved in the synthesis of the same or chemically similar non-canonical bases. Additional analysis of genomes in larger number also confirmed this. As known, bacteriophage T4 is not capable of general transduction (Wilson et al., 1979); moreover, it is excluded from infection by bacteriophage RB69, which has another modification of ^hm^dC (Russell, 1967). It was also shown in Wilson et al. (1979) that the frequency of transduction changed depending on associated proteins the phage had. Given that some of these proteins affect the host’s DNA, it can be assumed that these proteins will also change the frequency of other HGT events. These facts and our results allow us to suppose that non-canonical bases and the associated proteins formed a barrier for the exchange of genetic information between the related phages, which resulted in their divergence in the process of evolution.

It was also found that genes of the synthesis of non-canonical bases of ^hm^dC and its modifications (Figure 6) as well as genes of the synthesis of 7-deazaguanine modifications are located close to each other (most of them in one genome region). We speculate that genes of the synthesis of ^hm^dC and its modifications was a genome island able to be transferred horizontally. More detailed information on this event is given in Supplementary Data Sheet 5.

Taking into account that non-canonical bases and associated proteins are considered to be one of the mechanisms regulating the frequency of HGT in *Tevenvirinae*, and based on the results obtained in our study, it is supposed that they play one of the key roles in the genome diversity of these viruses.

Another important function of non-canonical bases is protection of phage DNA from the harm that can be inflicted by the systems of host bacteria restricting the development of phages. Bacteria possess systems of restriction-modification (R-M systems), which, in some cases, cleave canonical, and in other cases, non-canonical DNA. In addition, they have Cas systems, which can be inhibited by non-canonical bases – with the inhibition depending on the composition of the base set (Weigele and Raleigh, 2016; Vlot et al., 2018; Hutinet et al., 2019). In *Tevenvirinae*, hmdC and its modifications are known to have different protection against the restriction activity of R-M systems, and this is true for Cas systems as well (Bair and Black, 2007; Weigele and Raleigh, 2016; Hutinet et al., 2019). The phages containing 7-deazaguanine have also been demonstrated to have protection against R-M systems (Hutinet et al., 2019). Moreover, representatives of groups I–III possess IpI (Bair et al., 2007) and Arn (Kim et al., 1997) proteins (Table 2 and Supplementary Table 3), which protect them from the actions of type-IV R-M systems (these systems specifically affect DNA with non-canonical bases) (Weigele and Raleigh, 2016). Thus, the existence of multiple protective mechanism against anti-viral host systems erects another barrier to the exchange of genetic information between closely related phages, which increases the divergence of *Tevenvirinae*.

Summarizing the results of this work and the data on the barrier functions of non-canonical bases and associated proteins, we can envision a possible chain of events that led to the divergence of the genomes of *Tevenvirinae* ancestors. The first step was infection of various host bacteria: *Vibrio*, *Aeromonas*, *Enterobacteriales*, etc., by closely related phages – and, as a result, similar phages are widely distributed in various types of habitats: marine environments, soil, plants, and animals. Adaptation to the specific conditions of the habitats implied changes in the genomes of phages and host bacteria, which would allow the development of specific metabolic pathways. The ecological niches inhabited by *Tevenvirinae* phages vary greatly in terms of spatial barriers confining them from the environment – and so do the capabilities of the phages to HGT. In marine bacteriophages, HGT is facilitated; in phages inhabiting the gastrointestinal tract of animals and humans, it is limited. The differences in HGT may be reflected in the sizes of *Tevenvirinae* genomes (Petrov et al., 2010): the representatives isolated from marine sources are characterized by larger genomes as compared to the phages from other types of habitats – this being associated with the necessity of their ancestors to transfer a larger number of genes. The spatial isolation of bacteriophages and their adaptation to ecological niches played the greatest role in the divergence of ancestral phage genomes.

The second step was the appearance of various non-canonical bases in the DNA of *Tevenvirinae* ancestors. This event played a key role in the divergence of phage genomes within the same ecological niche. Phages with different sets of non-canonical bases and their modifications became more and more specialized in the infection of closely related bacteria. Next, the ancestors of *Tevenvirinae* inhabiting the same ecological niches started to diverge in the ways of exchange of genetic information. Some of them retained the ability to exchange genetic information with the organisms whose DNA has a canonical composition of bases. Others began to acquire genes whose products hindered HGT. As a result, the genomes of such phages became more conservative. Furthermore, the emergence of genes whose products protected non-canonical phage DNA from the action of type IV R-M systems expanded the capabilities of those phages to infect bacteria. The differences in the HGT potential also affected the size of the phage genomes. An example are representatives of groups XV and XVI, which have larger genomes as compared to the representatives of group I (Petrov et al., 2010).

As a matter of fact, the use of such a strategy for the exchange of genetic information is one of the reasons for the spread of *Tevenvirinae* in a variety of ecological niches. On the one hand, it allows them to acquire genes whose products are necessary for the survival in certain environments. On the other hand, it allows them to preserve these genes in their genomes. Under the pressure of natural selection, the populations, in which HGT is less constrained, will have an inherent advantage for propagating their offspring. However, as they became more and more adapted to the specific environmental conditions, the populations, in which HGT is limited, will have an advantage in the conservation of genes that are most critical for survival in a particular ecological niche. Eventually, the populations that manage to conserve the “best” set of survival-related genes will spread within the niche – yet, at the same time, will become less capable of spreading in other niches. In other words, the history of the evolutionary development of *Tevenvirinae* reflects their gradual specialization to the specific environments, with non-canonical bases and the associated proteins playing their part in the process of specialization.

It is difficult, if not impossible, to observe the full cycle of this evolutionary strategy of *Tevenvirinae* in every ecological niche, since the first and second steps would occur simultaneously and depend on each other. This is determined by the barrier properties of various habitats. As mentioned above, marine environments have the least number of barriers that would restrict the interaction of bacteria and phages. The total size of genetic pool of marine populations is large, and the flow of genetic information between different organisms is quite intense. Correspondingly, it would take more time for viruses to conserve a set of genes allowing them to excel in those habitats. In the ecological niches with higher barriers, the total size of genetic pool of populations is smaller, and the competition between different organisms is more severe. As a result, the rate of adaptation of viruses to the specific conditions of particular niches increases. We suppose that this strategy of *Tevenvirinae*, which is based on the restriction of genetic information exchange, may also be found in viruses of other taxonomic groups, inhabiting other niches. Taking into account the fact that the genomic islands associated with the synthesis of DNA bases are quite ancient, the chance to find viruses with such an evolutionary strategy is quite high.

The strategy of *Tevenvirinae* ancestors, making the exchange of genetic information dependent on the set of non-canonical bases and the associated proteins, was one of the main factors driving the spread of these phages across the environments – as indicated by their frequent occurrence in the metagenomes isolated from various sources.

Also, this strategy correlates with the Constant-Diversity (C-D) model (Mizuno et al., 2014). The model implies the co-existence of competing phage populations in the same biological niche, where each population is comprised by clonal lineages which differ in the presence of certain genes (metaviromic islands). Proteins encoded by such genes may, for example, adjust phage specificity to the host clonal lineages (Mizuno et al., 2014). The co-existence of different bacterial clonal lineages enriches the genetic pool of the bacterial population, and the comparable diversity of phages that prey on those bacteria has a stabilizing effect on it. When applied to our hypothesis, this leads to the conclusion that ancestral *Tevenvirinae* phages may have used different non-canonical bases to enable their development in the host cells which differed, for example, in genes encoding restriction-modification proteins.

Bacteriophages from other taxonomic groups that have non-canonical DNA bases may also use this strategy. If a sufficient number of phages of certain taxa is registered in the databases, the methods of pan-genomic analysis can be used to reveal the evolutionary strategies of these phages. To be handled efficiently, this task will also require the application of newly developed sequencing techniques. A comprehensive study of metabolism of viruses with non-canonical DNA bases, which would make use, alongside other approaches, the methods of comparative genomics, will contribute to the better understanding of the evolutionary mechanisms underlying the development of such viruses.

Nowadays, *Tevenvirinae* and a wider group of T4-related phages (Near-T4-related and Far-T4-related) represent an optimal model for studying the influence of non-canonical bases, their hypermodifications and the associated proteins on the evolution and ecology of viruses.

## Data Availability Statement

The original contributions presented in the study are included in the article/Supplementary Material, further inquiries can be directed to the corresponding authors.

## Author Contributions

NN and AZ designed the study, contributed to revision, and read and approved the final manuscript. NN organized the data, performed the data analysis, and drafted the manuscript. AZ conceived the study and critically revised the manuscript. Both authors contributed to the article and approved the submitted version.

## Conflict of Interest

The authors declare that the research was conducted in the absence of any commercial or financial relationships that could be construed as a potential conflict of interest.

## Supplementary Material

Supplementary Figure 1 Heat map representations of a number of homologous clusters in separate genomes. Horizontal lines represent genomes of viruses, vertical lines are homologous clusters.

Supplementary Figure 2 The pan-genomic tree of *Tevenvirinae* (shown in Figure 1) with labeled aBayes support/ultrafast bootstrap support. Black circles indicate the root and the nodes of branches with the same sequences as leaves.

Supplementary Figure 3 The phylogenetic tree of *Tevenvirinae* (presented in Figure 2) with labeled SH-aLRT support/aBayes support/ultrafast bootstrap support. Black circles indicate the root and the nodes of branches with the same sequences as leaves.

Supplementary Figure 4 The phylogenomic tree of *Tevenvirinae* (shown in Figure 3) with labeled GBDP pseudo-bootstrap support. Black circles indicate the root and the nodes of branches with the same sequences as leaves.

Supplementary Figure 5 The pan-genomic tree of *Tevenvirinae* extended genome set with labeled aBayes support/ultrafast bootstrap support. Selective clades are marked with Roman numerals and colors. Black circles indicate the root and the nodes of branches with the same sequences as leaves.

Supplementary Figure 6 The phylogenomic tree of *Tevenvirinae* extended genome set with labeled GBDP pseudo-bootstrap support. Selective clades are marked with Roman numerals and colors. Black circles indicate the root and the nodes of branches with the same sequences as leaves.

Supplementary Table 1 Supplementary data about studied genomes.

Supplementary Table 2 Supplementary data about studied GSCs.

Supplementary Table 3 Homologs of proteins associated with non-canonical bases in the studied genomes of *Tevenvirinae*.

Supplementary Table 4 GenBank accession numbers and IGM/VR Scaffolds IDs of genomes added for Additional Analysis.

Supplementary Data Sheet 1 Clustering results (folders “first clusterisation,” “second clusterisation,” and “third clusterisation”): the number of pan-genome components in the individual genome (files “pan genome components of genomes”), the total number of all clusters and components of the pan-genome (files “pan genome components”), a list of clusters of the core genome (files “core list”), a list of clusters of the softcore genome (files “softcore list”). Clusters of the softcore genome (folders “softcore clusters”) are presented in the files which show regions in genomes, protein sequences, names of viruses which have these proteins A description of softcore clusters is also supplied (file “Softcore clusters description”).

Supplementary Data Sheet 2 The results of the BLAST-assisted (“txt” files) and HMMER-assisted (“xlsx” files) searches for GSC homologs. The clusters containing homologs of proteins from other organisms are listed.

Supplementary Data Sheet 3 Clusters of the extended set of *Tevenvirinae* genomes. Lists of GSCs with homologs are presented (folder “GSCs”), which are described in Supplementary Table 2. Also, clusters of protein homologs associated with the synthesis of non-canonical bases are presented (folder “Non-canonical base clusters”), which are described in Table 2.

Supplementary Data Sheet 4 Characteristics of *Tevenvirinae* groups.

Supplementary Data Sheet 5 Characteristics of the region of genes of the synthesis of ^hm^dC and its modifications of *Tevenvirinae*.
