# Corrigendum: Influence of Non-canonical DNA Bases on the Genomic Diversity of *Tevenvirinae*

**DOI:** 10.3389/fmicb.2021.692581

**Published:** 2021-05-07

**Authors:** Nikita A. Nikulin, Andrei A. Zimin

**Affiliations:** ^1^Laboratory of Bacteriophage Biology, G.K. Skryabin Institute of Biochemistry and Physiology of Microorganisms, Pushchino Scientific Center for Biological Research of the Russian Academy of Sciences, Pushchino, Russia; ^2^Laboratory of Molecular Microbiology, G.K. Skryabin Institute of Biochemistry and Physiology of Microorganisms, Pushchino Scientific Center for Biological Research of the Russian Academy of Sciences, Pushchino, Russia

**Keywords:** *Tevenvirinae*, phages, non-canonical bases, modified bases, genome diversity

In the original article, there was an error. The sentence “Since the region is surrounded by the genes of helicase and DNA polymerase, we have analyzed the genome fragments between them.” contain a mistake in the name of one of the genes between which the region is located.

A correction has been made to the Results section, subsection Non-canonical Bases and Related Proteins, Paragraph 3:

Since the region is surrounded by the genes of head vertex assembly chaperone and DNA polymerase, we have analyzed the genome fragments between them.

Additionally, there was a similar mistake in the legend for **Figure 6** as published. Head vertex assembly chaperone gene was mistakenly named helicase gene. The correct legend appears below.

**FIGURE 6 |** Variations in the region between the genes homologous to the genes of DNA polymerase and head vertex assembly chaperone in closely related *Tevenvirinae* that have homologs of ^hm^dC-associated proteins. The genes whose products are described in Table 2 are indicated by color; the genes whose products have homologs with known functions are additionally marked.

The sentence “It should be noted that in Acinetobacter phage Acj9, the genes whose products are supposedly associated with the synthesis of non-canonical bases are located in a wider region of the genome: between the genes of sliding clamp loader proteins and DNA polymerase (a part of the core genome)” contain a mistake in the name of one of the genes between which the region is located.

A correction has been made to the Results section, subsection Non-canonical Bases and Related Proteins, Paragraph 4:

It should be noted that in Acinetobacter phage Acj9, the genes whose products are supposedly associated with the synthesis of non-canonical bases are located in a wider region of the genome: between the genes of helicase proteins and DNA polymerase (a part of the core genome).

Additionally, a similar mistake was made in Supplementary Data Sheet 5. Helicase gene was mistakenly named sliding clamp loader gene. Corrections have been made by replacing Supplementary Data Sheet 5 with a file with the correct gene name.

The authors apologize for these errors and state that they do not change the scientific conclusions of the article in any way. The original article has been updated.

