# Supplementary figures and images for: Influence of Non-canonical DNA Bases on the Genomic Diversity of Tevenvirinae
Source: Front Microbiol. 2021 Apr 6;12:632686. doi: 10.3389/fmicb.2021.632686 (PMC8056088; doi:10.3389/fmicb.2021.632686)

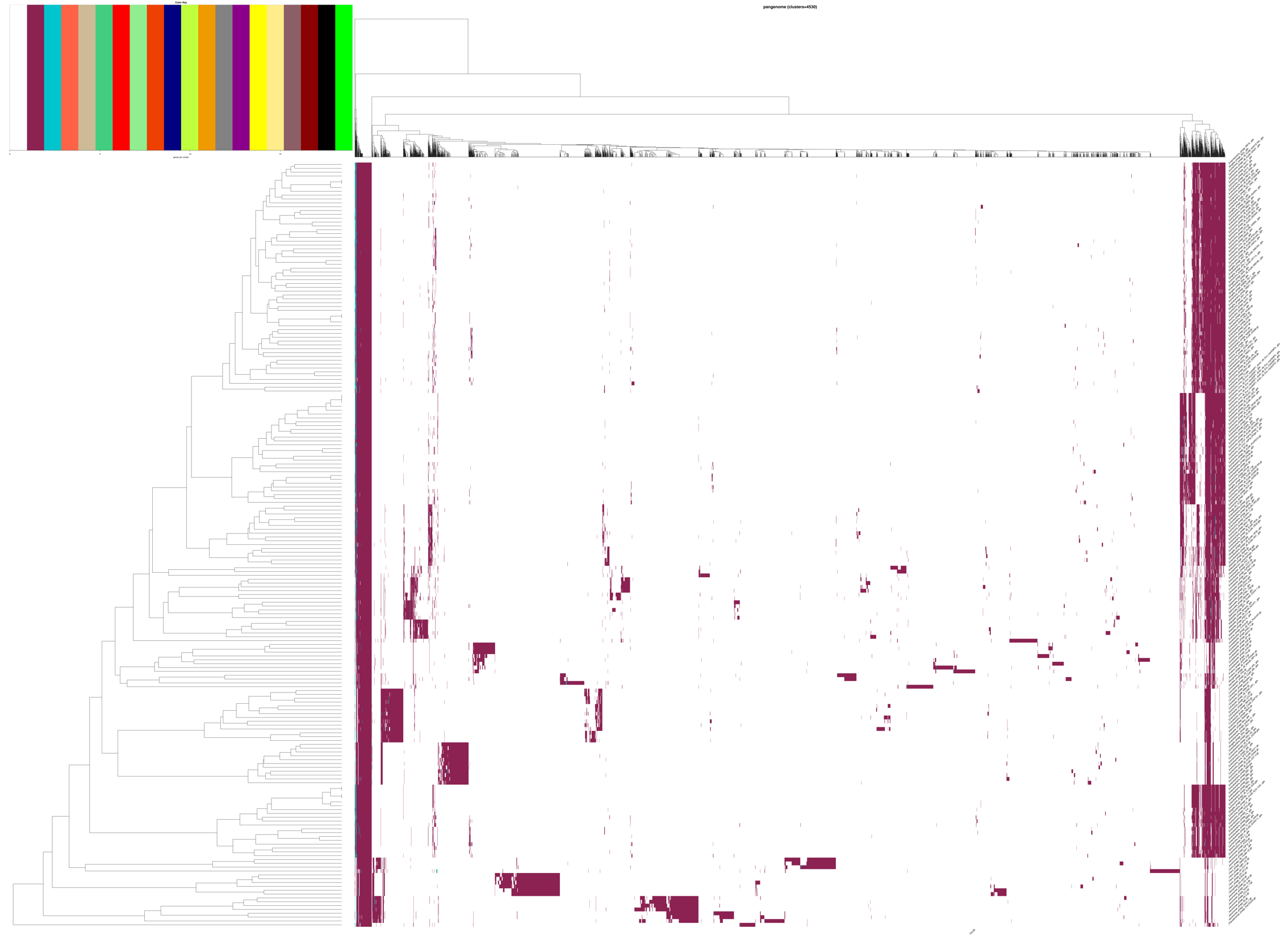

Supplement: Supplementary Figure 1 — Heat map representations of a number of homologous clusters in separate genomes. Horizontal lines represent genomes of viruses, vertical lines are homologous clusters. [file Image_1.PDF]

label

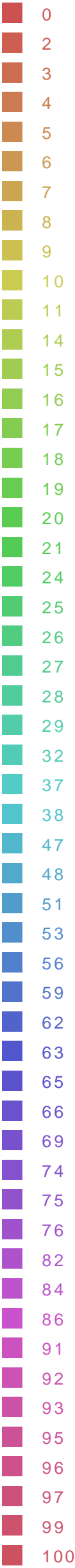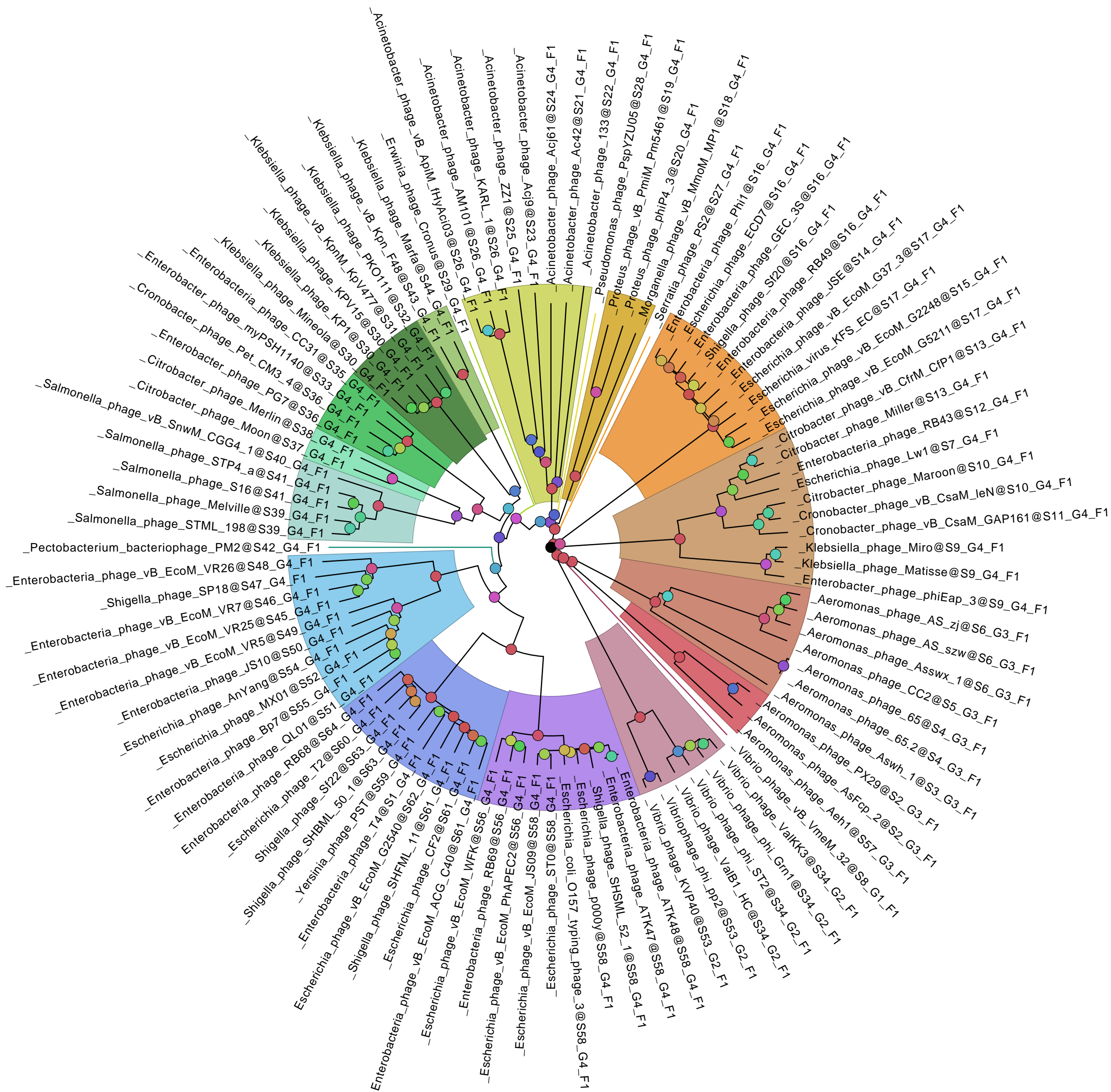

Supplement: Supplementary Figure 4 — The phylogenomic tree of Tevenvirinae (shown in Figure 3) with labeled GBDP pseudo-bootstrap support. Black circles indicate the root and the nodes of branches with the same sequences as leaves. [file Image_4.PDF]

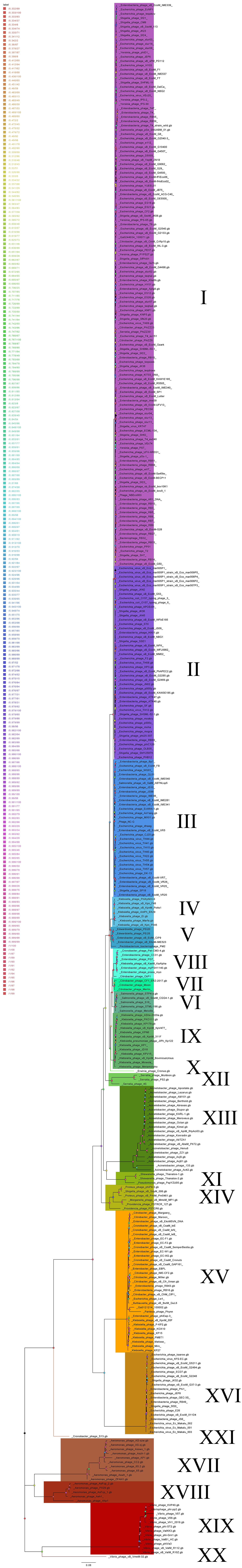

Supplement: Supplementary Figure 5 — The pan-genomic tree of Tevenvirinae extended genome set with labeled aBayes support/ultrafast bootstrap support. Selective clades are marked with Roman numerals and colors. Black circles indicate the root and the nodes of branches with the same sequences as leaves. [file Image_5.TIF]

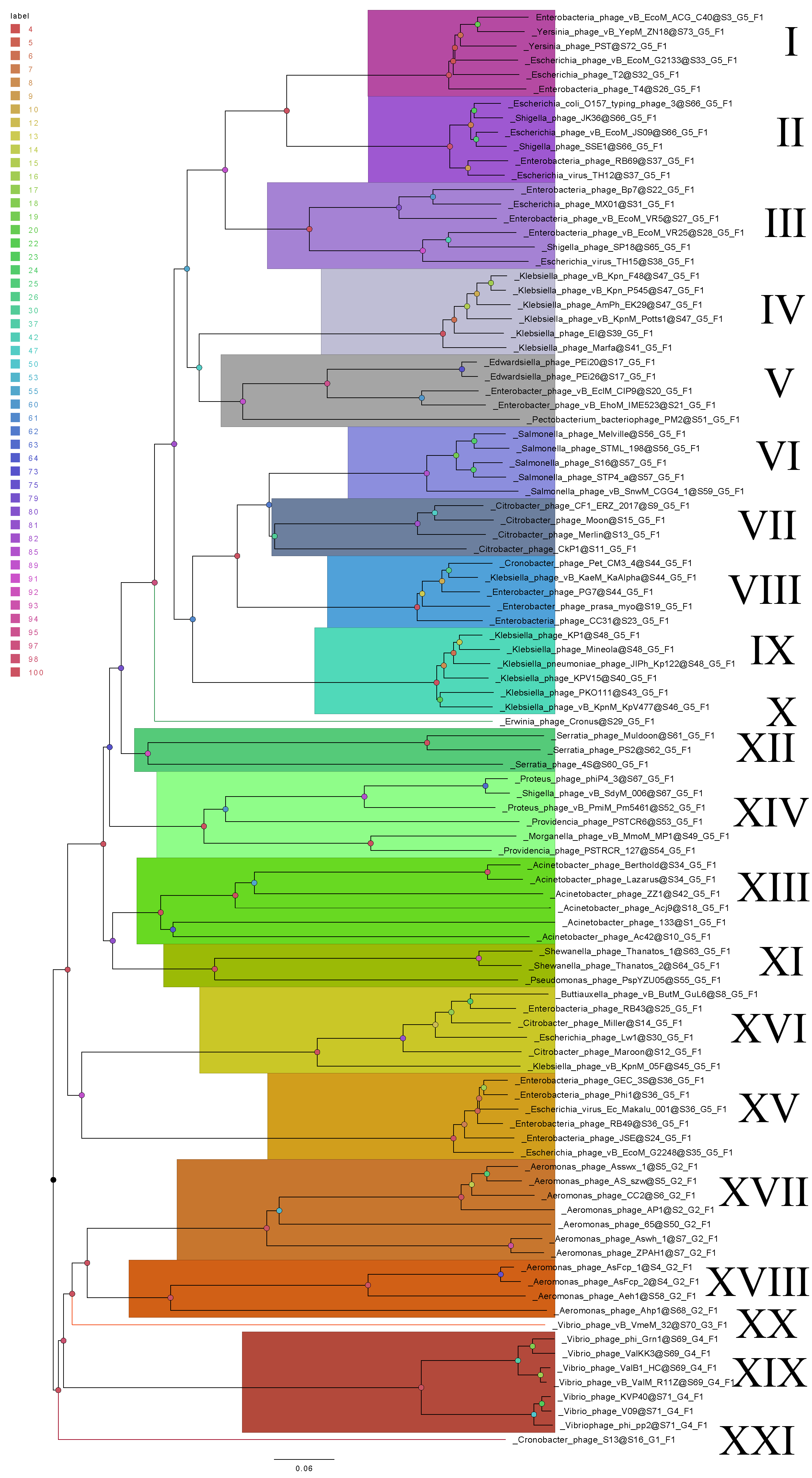

Supplement: Supplementary Figure 6 — The phylogenomic tree of Tevenvirinae extended genome set with labeled GBDP pseudo-bootstrap support. Selective clades are marked with Roman numerals and colors. Black circles indicate the root and the nodes of branches with the same sequences as leaves. [file Image_6.TIF]

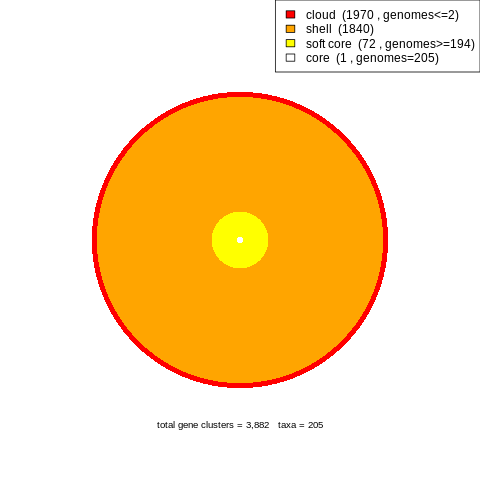

Supplement: Supplementary Data Sheet 1 — Clustering results (folders “first clusterisation,” “second clusterisation,” and “third clusterisation”): the number of pan-genome components in the individual genome (files “pan genome components of genomes”), the total number of all clusters and components of the pan-genome (files “pan genome components”), a list of clusters of the core genome (files “core list”), a list of clusters of the softcore genome (files “softcore list”). Clusters of the softcore genome (folders “softcore clusters”) are presented in the files which show regions in genomes, protein sequences, names of viruses which have these proteins A description of softcore clusters is also supplied (file “Softcore clusters description”). [file Data_Sheet_1.ZIP › Data sheet 1/first clusterisation/pan_genome_components.png]

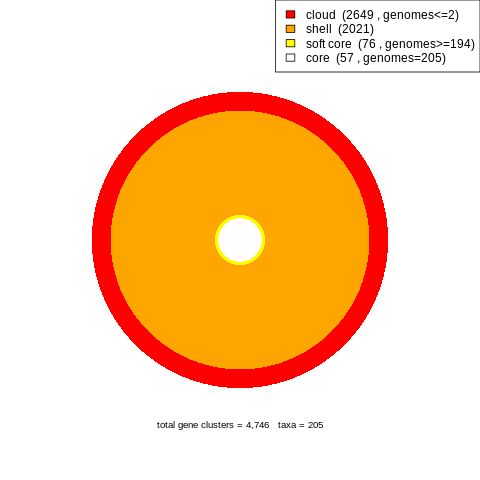

Supplement: Supplementary Data Sheet 1 — Clustering results (folders “first clusterisation,” “second clusterisation,” and “third clusterisation”): the number of pan-genome components in the individual genome (files “pan genome components of genomes”), the total number of all clusters and components of the pan-genome (files “pan genome components”), a list of clusters of the core genome (files “core list”), a list of clusters of the softcore genome (files “softcore list”). Clusters of the softcore genome (folders “softcore clusters”) are presented in the files which show regions in genomes, protein sequences, names of viruses which have these proteins A description of softcore clusters is also supplied (file “Softcore clusters description”). [file Data_Sheet_1.ZIP › Data sheet 1/second clusterisation/pan_genome_components.png]

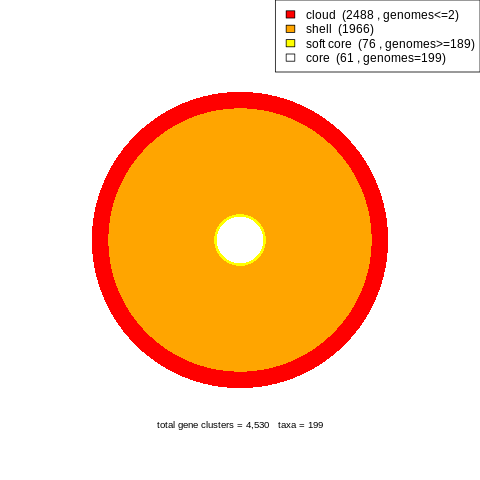

Supplement: Supplementary Data Sheet 1 — Clustering results (folders “first clusterisation,” “second clusterisation,” and “third clusterisation”): the number of pan-genome components in the individual genome (files “pan genome components of genomes”), the total number of all clusters and components of the pan-genome (files “pan genome components”), a list of clusters of the core genome (files “core list”), a list of clusters of the softcore genome (files “softcore list”). Clusters of the softcore genome (folders “softcore clusters”) are presented in the files which show regions in genomes, protein sequences, names of viruses which have these proteins A description of softcore clusters is also supplied (file “Softcore clusters description”). [file Data_Sheet_1.ZIP › Data sheet 1/third clusterisation/pan_genome_components.png]
