# Supplementary material for: Influence of Non-canonical DNA Bases on the Genomic Diversity of Tevenvirinae
Source: Front Microbiol. 2021 Apr 6;12:632686. doi: 10.3389/fmicb.2021.632686 (PMC8056088; doi:10.3389/fmicb.2021.632686)
Supplement: Supplementary Table 1 — Supplementary data about studied genomes. [file Table_1.PDF]

| ID | Phages | Sensitive bacteria* | Sources* | References |
|---|---|---|---|---|
| NC_015250.1 | Acinetobacter phage 133 | *A. johnsonii* (HER 1423) | raw sewage, Quebec, Canada | 1,2, 3 |
| NC_014660.1 | Acinetobacter phage Ac42 | *Acinetobacter sp.* | therapeutic phage library, Eliava Institute of Bacteriophage, Georgia | 3 |
| NC_014661.1 | Acinetobacter phage Acj61 | *A. johnsonii* | sewage, New Orlean, USA | 3 |
| NC_014663.1 | Acinetobacter phage Acj9 | *A. johnsonii* | sewage, New Orlean, USA | 3 |
| MH165274.1 | Acinetobacter phage AM101 | *A. baumannii* LUH 3712 | water, river Moscow, Russia | - |
| MH713599.1 | Acinetobacter phage KARL-1 | *A. baumannii* | pond water, Germany | 4 |
| MH460829.1 | Acinetobacter phage vB_ApiM_fHyAci03 | *A. pittii* | municipal sewage, Hyvinkää, Finland | 5 |
| NC_018087.3 | Acinetobacter phage ZZ1 | *A.baumannii* | sewage, fishpond along the Yellow River, China | 6,7 |
| NC_015251.1 | Aeromonas phage 65 | *A. salmonicida* | water, river “La Petite Mouge”, France | 1, 3, 8, 9 |
| KY290955.1 | Aeromonas phage 65.2 | *A. salmonicida* | water, river “La Petite Mouge”, France | 1, 3, 8, 9, 10 |
| NC_005260.1 | Aeromonas phage Aeh1 | *A. hydrophila* | sewage, Oshkosh, USA | 3, 11, 12, 13 |
| MG250483.1 | Aeromonas phage Ah1 | *A. hydrophila* | wastewater, Russia | - |
| MF498773.1 | Aeromonas phage AS-szw | *A. salmonicida* | water from river, Guangdong Province, China | 14 |
| MF448340.1 | Aeromonas phage AS-zj | *A. salmonicida* | water from river, Guangdong Province, China | 14 |
| MH791401.1 | Aeromonas phage AsFcp_2 | *A. salmonicida* | sewage | - |
| MH791398.1 | Aeromonas phage Asswx_1 | *A. salmonicida* | sewage | - |
| MH791414.1 | Aeromonas phage Aswh_1 | *A. salmonicida* | freshwater | - |
| NC_019538.1 | Aeromonas phage CC2 | *A.hydrophila* | sewage, China | 15 |
| NC_014636.1 | Aeromonas phage phiAS5 | *A. salmonicida* | water, Han-river, Korea | 16 |
| NC_023688.1 | Aeromonas phage PX29 | *A. salmonicida* | sewage, New Orlean, USA | 3 |
| NC_008515.1 | Bacteriophage RB32 | *E.coli* | sewage treatment plants, Long Island, USA | 17 |
| NC_029013.1 | Citrobacter phage IME-CF2 | *C. freundii* | hospital sewage, China | - |
| NC_028755.1 | Citrobacter phage Margaery | *C. freundii* | - | - |
| MH823906.1 | Citrobacter phage Maroon | *C. freundii* | wastewater, Brazos County, USA | 18 |
| NC_028857.1 | Citrobacter phage Merlin | *C. freundii* | water, College Station, USA | 19 |
| NC_025414.1 | Citrobacter phage Miller | *C. freundii* | sewage, Bryan, USA | 20 |
| NC_027331.1 | Citrobacter phage Moon | *C. freundii* | sewage, College Station, USA | 21 |
| NC_031057.1 | Citrobacter phage vB_CfrM_CfP1 | *C. freundii* | wastewater treatment plant, Frossos, Portugal | 22 |
| LT614807.1 | Cronobacter phage Pet-CM3-4 | *Cr. malonaticus* | wastewater treatment plant, Petrzalka, Slovakia | 23 |

| | | | | |
|---|---|---|---|---|
| NC_019398.1 | Cronobacter phage vB_CsaM_GAP161 | *Cr. sakazakii* | sewage, Guelph, Canada | 24 |
| KX431559.1 | Cronobacter phage vB_CsaM_leB | *Cr. sakazakii* | slurry, cattle farmer in Clonakilty, Ireland | 25 |
| KX431560.1 | Cronobacter phage vB_CsaM_leN | *Cr. sakazakii* | slurry, cattle farmer in Clonakilty, Ireland | 25 |
| MG999954.1 | Enterobacter phage myPSH1140 | *E. cloacae* | sewage water treatment plants, India | 26 |
| NC_023561.1 | Enterobacter phage PG7 | *E. cloacae* | fishpond water, China | - |
| NC_041980.1 | Enterobacter phage phiEap-3 | *E. aerogenes* | sewage wastewater sample, Navy General Hospital, China | 27 |
| KT184308.1 | Enterobacteria phage Aplg8 | *E. coli* O121:H19 | sewage, USA | - |
| NC_027983.1 | Enterobacteria phage AR1 DNA | *E. coli* O157:H7 | fresh manure, Dairy farms in St. Croix County , USA | 28 |
| KT184309.1 | Enterobacteria phage ATK47 | *E. coli* O26:H11 | sewage, USA | - |
| KT184310.1 | Enterobacteria phage ATK48 | *E. coli* O26:H12 | sewage, USA | - |
| NC_019500.1 | Enterobacteria phage Bp7 | *E. coli* O78.A | chicken feces, Shandong Province, China | 29 |
| NC_014662.1 | Enterobacteria phage CC31 | *E. coli* B strain S/6/4 | sewage, New Orlean, USA | 3 |
| NC_025425.1 | Enterobacteria phage GEC-3S | *E. coli* O104:H4 | therapeutic phage library, Eliava Institute of Bacteriophage, Georgia | 30 |
| KT184311.1 | Enterobacteria phage GiZh | *E. coli* O111:NM 96-3166 | sewage, USA | - |
| NC_018855.1 | Enterobacteria phage HX01 | *E. coli* DM01 | duck feces, duck factory, China | 31 |
| NC_014260.1 | Enterobacteria phage IME08 | *E. coli* strain 8099 | hospital sewage, China | 32 |
| NC_012741.1 | Enterobacteria phage JS10 | *E. coli* K12 | feces, diarrhea patient, Bangladesh | 33 |
| NC_010105.1 | Enterobacteria phage JS98 | *E. coli* | feces, diarrhea patient, Bangladesh | 33 |
| NC_012740.1 | Enterobacteria phage JSE | *E.coli* K12 | sewage, sewage station, Switzerland | 33 |
| KT184312.1 | Enterobacteria phage Kha5h | *E. coli* O103:H2 90-3128 | sewage, USA | - |
| NC_009821.1 | Enterobacteria phage Phi1 | *E. coli* K-12 (F+) | therapeutic phage library, Eliava Institute of Bacteriophage, Georgia | 34 |
| NC_028847.1 | Enterobacteria phage QL01 | *E. coli* DE205B | duck feces, poultry market in Nanjing, China | 67 |
| KM606999.1 | Enterobacteria phage RB10 | *E.coli* | sewage treatment plants, Long Island, USA | 17 |
| NC_012638.1 | Enterobacteria phage RB14 | *E.coli* | sewage treatment plants, Long Island, USA | 17 |
| NC_014467.1 | Enterobacteria phage RB16 | *E.coli* | sewage treatment plants, Long Island, USA | 17 |
| MH553563.1 | Enterobacteria phage RB18 | *E.coli* | sewage treatment plants, Long Island, USA | 17 |
| NC_025448.1 | Enterobacteria phage RB27 | *E.coli* | sewage treatment plants, Long Island, USA | 17 |
| NC_025419.1 | Enterobacteria phage RB3 | *E.coli* | sewage treatment plants, Long Island, USA | 17 |
| KM607001.1 | Enterobacteria phage RB33 | *E.coli* | sewage treatment plants, Long Island, USA | 17 |
| NC_007023.1 | Enterobacteria phage RB43 | *E.coli* | sewage treatment plants, Long Island, USA | 17 |
| NC_005066.1 | Enterobacteria phage RB49 | *E.coli* | sewage treatment plants, Long Island, USA | 17 |
| KM606995.1 | Enterobacteria phage RB5 | *E.coli* | sewage treatment plants, Long Island, USA | 17 |

| | | | | |
|---|---|---|---|---|
| NC_012635.1 | Enterobacteria phage RB51 | *E.coli* | sewage treatment plants, Long Island, USA | 17 |
| KM607002.1 | Enterobacteria phage RB55 | *E.coli* | sewage treatment plants, Long Island, USA | 17 |
| KM607003.1 | Enterobacteria phage RB59 | *E.coli* | sewage treatment plants, Long Island, USA | 17 |
| KM606996.1 | Enterobacteria phage RB6 | *E.coli* | sewage treatment plants, Long Island, USA | 17 |
| NC_027979.1 | Enterobacteria phage RB68 | *E.coli* | sewage treatment plants, Long Island, USA | 17 |
| NC_004928.1 | Enterobacteria phage RB69 | *E.coli* | sewage treatment plants, Long Island, USA | 17 |
| KM606997.1 | Enterobacteria phage RB7 | *E.coli* | sewage treatment plants, Long Island, USA | 17 |
| KM606998.1 | Enterobacteria phage RB9 | *E.coli* | sewage treatment plants, Long Island, USA | 17 |
| NC_000866.4 | Enterobacteria phage T4 | *E.coli* | sewage/ fecal material | 36,37 |
| KJ477684.1 | Enterobacteria phage T4 strain wild | *E.coli* | sewage/ fecal material | 36, 37, 38 |
| HM137666.1 | Enterobacteria phage T4T | *E.coli* | sewage/ fecal material | 36,37 |
| MH550421.1 | Enterobacteria phage T6 | *E.coli* | sewage/ fecal material | 36,37 |
| NC_019399.1 | Enterobacteria phage vB_EcoM_ACG-C40 | *E.coli* | sewage, Guelph water treatment plant, Canada | 39 |
| MH051915.1 | Enterobacteria phage vB_EcoM_IME339 | *E. coli* BL21(DE3) | sewage, State Key Laboratory of Pathogens and Biosecurity, China | 40 |
| MH051916.1 | Enterobacteria phage vB_EcoM_IME340 | *E. coli* BL21(DE3) | sewage, State Key Laboratory of Pathogens and Biosecurity, China | 40 |
| NC_028894.1 | Enterobacteria phage vB_EcoM_VR20 | *E.coli* Be | municipal wastewater, Lithuania | 41 |
| NC_028925.1 | Enterobacteria phage vB_EcoM_VR25 | *E. coli* MH1 | municipal wastewater, Lithuania | 42 |
| NC_028957.1 | Enterobacteria phage vB_EcoM_VR26 | *E. coli* MH1 | municipal wastewater, Lithuania | 42 |
| NC_028881.1 | Enterobacteria phage vB_EcoM_VR5 | *E.coli* Be | sewage, Lithuania | 41 |
| NC_014792.1 | Enterobacteria phage vB_EcoM-VR7 | *E.coli* Be | sewage, Lithuania | 41 |
| MH059636.2 | Erwinia phage Cronus | *Er. amylovora* DSM17948 | organic waste, Denmark | - |
| NC_041863.1 | Escherichia coli O157 typing phage 3 | *E. coli* O157:H7 | National Microbiology Laboratory, Winnipeg | 43 |
| NC_041864.1 | Escherichia coli O157 typing phage 6 | *E. coli* O157:H7 | National Microbiology Laboratory, Winnipeg | 43 |
| MK234886.1 | Escherichia phage AnYang | *E. coli* O157 | soil, China | - |
| NC_029091.1 | Escherichia phage APCEc01 | *E. coli* DPC6051 | human faecal, Ireland | 44 |

| | | | | |
|---|---|---|---|---|
| NC_041919.1 | Escherichia phage CF2 | *E. coli* CP9 | chicken feces, Texas private farm, USA | 45 |
| MK327929.1 | Escherichia phage D5505 | *E. coli* DSM101114 | surface water, Braunschweig, Germany | 46 |
| NC_024125.2 | Escherichia phage e11/2 | *E.coli* O157:H7 | bovine fecal, bovine fecal, Ireland | 47,48 |
| NC_041936.1 | Escherichia phage ECD7 | *E. coli* O104:H4 | chicken excrements, Moscow region, Russia | 50,49 |
| NC_025449.1 | Escherichia phage ECML-134 | *E. coli* O157:H7 | fresh and salt water, water estuary, Georgia | 51 |
| MH791409.1 | Escherichia phage EcWhh-1 | *E.coli* | freshwater | - |
| NC_041920.1 | Escherichia phage HP3 | *E. coli* CP9 | duck/geese feces, Houston parks, USA | 45 |
| NC_027349.1 | Escherichia phage HY01 | *E. coli* O157:H7 | swine fecal, farm at Seoul National University in Suwon, South Korea | 52 |
| NC_031047.1 | Escherichia phage HY03 | *Escherichia coli* O157:H7 | - | - |
| AP018932.1 | Escherichia phage KIT03 | *Escherichia coli* O157:H7 | soil, Kyoto poultry farm, Japan | 53 |
| NC_021344.2 | Escherichia phage Lw1 | *Escherichia coli BL21*(DE3) | contaminant of lysed cells | 54 |
| NC_031934.1 | Escherichia phage MX01 | *Escherichia coli* DE217 | duck feces | - |
| MH992122.1 | Escherichia phage OLB35 | *Escherichia coli* | wastewater treatment facility | - |
| MK047717.1 | Escherichia phage p000v | *Escherichia coli* O157:H7 | wastewater, San Francisco, California | 55 |
| MK047718.1 | Escherichia phage p000y | *Escherichia coli* O157:H7 | wastewater, San Francisco, California | 55 |
| KU925172.1 | Escherichia phage PE37 | *Escherichia coli* O157:H7 | bovine intestine, retail stores in Fukuoka, Japan | 100 |
| LC348379.1 | Escherichia phage PP01 | *Escherichia coli* O157:H7 | swine stool, Japan | 56 |
| MH359124.1 | Escherichia phage SF | *Escherichia coli* O157:H7 | sewage facility, Universiti Sains Malaysia campus in Penang, Malaysia | 57 |
| NC_028927.1 | Escherichia phage slur02 | *Escherichia coli* MG1655 | slurry, dairy farm slurry tank in the East Midlands, UK | 58 |
| NC_042129.1 | Escherichia phage slur03 | *Escherichia coli* MG1655 | slurry, dairy farm slurry tank in the East Midlands, UK | 58 |
| NC_042130.1 | Escherichia phage slur04 | *Escherichia coli* MG1655 | slurry, dairy farm slurry tank in the East Midlands, UK | 58 |
| NC_028780.1 | Escherichia phage slur07 | *Escherichia coli* MG1655 | slurry, dairy farm slurry tank in the East Midlands, UK | 58 |
| LN881733.1 | Escherichia phage slur08 | *Escherichia coli* MG1655 | slurry, dairy farm slurry tank in the East Midlands, UK | 58 |
| LN881734.1 | Escherichia phage slur11 | *Escherichia coli* MG1655 | slurry, dairy farm slurry tank in the East Midlands, UK | 58 |
| LN881737.1 | Escherichia phage slur13 | *Escherichia coli* MG1655 | slurry, dairy farm slurry tank in the East Midlands, UK | 58 |
| NC_028448.1 | Escherichia phage slur14 | *Escherichia coli* MG1655 | slurry, dairy farm slurry tank in the East Midlands, UK | 58 |
| NC_041990.1 | Escherichia phage ST0 | *Escherichia coli* H8 | wastewater, Beijing sewage treatment plant, China | 59 |
| LC348380.1 | Escherichia phage T2 | *E.coli* | sewage/ fecal | 36,37 |
| NC_031030.1 | Escherichia phage UFV-AREG1 | *Escherichia coli* O157 | cowshed wastewater, Brazil | 60 |
| MH992510.1 | Escherichia phage vB_EcoM_DalCa | *E.coli* | raw sewage, Frossos wastewater treatment plant, Portugal | - |
| MK327937.1 | Escherichia phage vB_EcoM_G10400 | *E.coli* DSM 103266 | pig manure, Hameln, Germany | 46 |

| | | | | |
|---|---|---|---|---|
| MK327932.1 | Escherichia phage vB_EcoM_G2248 | *E.coli* DSM 103255 | pig manure, Hameln, Germany | 46 |
| MK327933.1 | Escherichia phage vB_EcoM_G2285 | *E.coli* DSM 103256 | pig manure, Hameln, Germany | 46 |
| MK327934.1 | Escherichia phage vB_EcoM_G2469 | *E.coli* DSM 103258 | pig manure, Hameln, Germany | 46 |
| MK327935.1 | Escherichia phage vB_EcoM_G2494 | *E.coli* DSM 103259 | pig manure, Hameln, Germany | 46 |
| MK327936.1 | Escherichia phage vB_EcoM_G2540 | *E.coli* DSM 103260 | pig manure, Hameln, Germany | 46 |
| MK327944.1 | Escherichia phage vB_EcoM_G2540-3 | *E.coli* DSM 103260 | pig manure, Hameln, Germany | 46 |
| MK327940.1 | Escherichia phage vB_EcoM_G29 | *E.coli* DSM 103247 | pig manure, Hameln, Germany | 46 |
| MK327941.1 | Escherichia phage vB_EcoM_G37-3 | *E.coli* DSM 103248 | pig manure, Hameln, Germany | 46 |
| MK327939.1 | Escherichia phage vB_EcoM_G4498 | *E.coli* DSM 103261 | pig manure, Hameln, Germany | 46 |
| MK327945.1 | Escherichia phage vB_EcoM_G4500 | *E.coli* DSM 103262 | pig manure, Hameln, Germany | 46 |
| MK327946.1 | Escherichia phage vB_EcoM_G4507 | *E.coli* DSM 103263 | pig manure, Hameln, Germany | 46 |
| MK327942.1 | Escherichia phage vB_EcoM_G50 | *E.coli* DSM 103250 | pig manure, Hameln, Germany | 46 |
| MK327947.1 | Escherichia phage vB_EcoM_G5211 | *E.coli* DSM 103264 | pig manure, Hameln, Germany | 46 |
| MK327943.1 | Escherichia phage vB_EcoM_G53 | *E.coli* DSM 103251 | pig manure, Hameln, Germany | 46 |
| MK373787.1 | Escherichia phage vB_EcoM_G8 | *E.coli* DSM 103243 | pig manure, Hameln, Germany | 46 |
| MK373779.1 | Escherichia phage vB_EcoM_G9062 | *E.coli* DSM 103265 | pig manure, Hameln, Germany | 46 |
| MH355584.1 | Escherichia phage vB_EcoM_JB75 | *E.coli* | raw sewage, Frossos wastewater treatment plant, Portugal | 61 |
| NC_024124.2 | Escherichia phage vB_EcoM_JS09 | *Escherichia coli* (APEC) | sewage, swine farm in Jiangsu Province, China | 62 |
| MK373781.1 | Escherichia phage vB_EcoM_KAW1E185 | *E.coli* DSM 103253 | clinical wastewater, Braunschweig, Germany | 46 |
| MK373782.1 | Escherichia phage vB_EcoM_KAW3E185 | *E.coli* DSM 103253 | clinical wastewater, Braunschweig, Germany | 46 |
| MK373784.1 | Escherichia phage vB_EcoM_MM02 | *E.coli* DSM 498 | duck feces, Braunschweig, Germany | 46 |

| | | | | |
|---|---|---|---|---|
| MK373785.1 | Escherichia phage vB_EcoM_OE5505 | *E.coli* DSM 101114 | surface water, Braunschweig, Germany | 46 |
| NC_024794.1 | Escherichia phage vB_EcoM_PhAPEC2 | *Escherichia coli* (APEC) | water, rivers and brooks in and around Brussels in the vicinity of poultry houses, Belgium | 63 |
| MK373786.1 | Escherichia phage vB_EcoM_R5505 | *E.coli* DSM 101114 | surface water, Braunschweig, Germany | 46 |
| MK373778.1 | Escherichia phage vB_EcoM_WFbE185 | *E.coli* DSM 103253 | sewage, Wolfenbüttel, Germany | 46 |
| MK373775.1 | Escherichia phage vB_EcoM_WFK | *E.coli* DSM 101124 | sewage, Wolfenbüttel, Germany | 46 |
| MK373774.1 | Escherichia phage vB_EcoM_WFL6982 | *E.coli* DSM 101124 | sewage, Wolfenbüttel, Germany | 46 |
| MG781190.1 | Escherichia phage vB_EcoM-fFiEco06 | *Escherichia coli* 123738 | municipal wastewater, Finland | 64 |
| MG781191.1 | Escherichia phage vB_EcoM-fHoEco02 | *Escherichia coli* 123738 | hospital wastewater, Helsinki, Finland | 64 |
| MG867727.1 | Escherichia phage vB_EcoM-G28 | *E.coli* | manure, Germany | - |
| MK977694.1 | Escherichia phage vB_EcoM-Sa45lw | *Escherichia coli* ATCC 13706 | surface water, Salinas produce-growing area, USA | 65 |
| NC_031103.1 | Escherichia phage vB_EcoM-UFV13 | *Escherichia coli* UFV30 | sewage, Viçosa sewerage system, Brasil | 66, 101 |
| NC_031928.1 | Escherichia phage WG01 | *Escherichia coli* DE017 | duck feces, poultry market in Nanjing, China | 67 |
| NC_019505.1 | Escherichia phage wV7 | *Escherichia coli* O157:H7 | human feces/hamburger meat | 68,69 |
| MH560358.1 | Escherichia virus KFS-EC | *Escherichia coli* O157:H7 | wastewater, South Korea | - |
| LR027390.1 | Escherichia virus vB_Eco_mar005P1 | *E.coli* MG1655 | sea water, Great Yarmouth, UK | 70 |
| LR027387.1 | Escherichia virus vB_Eco_mar005P1 strain vB_Eco_mar006P2 | *E.coli* MG1655 | sea water, Great Yarmouth, UK | 70 |
| LR027383.1 | Escherichia virus vB_Eco_mar005P1 strain vB_Eco_mar007P3 | *E.coli* MG1655 | sea water, Great Yarmouth, UK | 70 |
| LR027386.1 | Escherichia virus vB_Eco_mar005P1 strain vB_Eco_mar008P4 | *E.coli* MG1655 | sea water, Great Yarmouth, UK | 70 |

| | | | | |
|---|---|---|---|---|
| LR027391.1 | Escherichia virus vB_Eco_mar005P1 strain vB_Eco_mar009P5 | *E.coli* MG1655 | sea water, Great Yarmouth, UK | 70 |
| MN022785.1 | Escherichia virus VEc20 | *Escherichia coli* | feces and sewage, poultry farm, Russia | - |
| MG751100.1 | Klebsiella phage KP1 | *Klebsiella pneumoniae* | sewage, Chungcheongbuk-do, South Korea | 71 |
| KY000080.1 | Klebsiella phage KPV15 | *Klebsiella pneumoniae* | clinical material, Russia | - |
| MN044033.1 | Klebsiella phage Marfa | *K. pneumoniae* 1776c | swine fecal, Texas and Michigan, USA | 72 |
| NC_028750.1 | Klebsiella phage Matisse | *Klebsiella pneumoniae* | sewage, College Station, USA | 73 |
| MH333064.1 | Klebsiella phage Mineola | *K. pneumoniae* | activated sludge, Bryan municipal wastewater, USA | 74 |
| NC_041981.1 | Klebsiella phage Miro | *K. pneumoniae* A1 | sewage, College Station, USA | 75 |
| NC_031095.1 | Klebsiella phage PKO111 | *Klebsiella oxytoca* ATCC 43863 | sewage, Suwon sewage treatment facility, South Korea | 76 |
| MG746602.1 | Klebsiella phage vB_Kpn_F48 | *K. pneumoniae* 12C47 | sewage, Florence Careggi University hospital, Italy | 77 |
| NC_031087.1 | Klebsiella phage vB_KpnM_KpV477 | *Klebsiella pneumoniae* KPB463 | clinical sample, Burdenko Neurosurgery Institute, Russia | 78 |
| KX078569.1 | Morganella phage vB_MmoM_MP1 | *M. morganii* | sewage, Braga wastewater treatment plant, Portugal | 79 |
| NC_028940.1 | Pectobacterium bacteriophage PM2 | *P. carotovorum* subsp. *carotovorum* | soil, Chinese cabbage fields in Pyeongchang, South Korea | 80 |
| MK310184.1 | Phage NC-G | *Escherichia coli* NC101 | human fecal, individuals diagnosed with Crohn’s disease, USA | 81 |
| MG696114.1 | Proteus phage phiP4-3 | *Proteus penneri* | fish, China | - |
| NC_028762.1 | Proteus phage vB_PmiM_Pm5461 | *Proteus mirabilis* 417 | wastewater plant raw effluent, Portugal | - |
| KY971610.1 | Pseudomonas phage PspYZU05 | *Pseudomonas sp.* | sewage | - |
| NC_042044.1 | Salmonella phage Melville | *Salmonella enterica* subsp. *enterica* serovar Newport | wastewater, Austin, USA | 82 |
| NC_020416.1 | Salmonella phage S16 | *S. enterica* | sewage, Gelderland municipal sewage plant, Netherlands | 83 |
| NC_027344.1 | Salmonella phage STML-198 | *Salmonella* | surface water, Maryland, USA | 84 |
| NC_026607.2 | Salmonella phage STP4-a | *Salmonella Typhimurium ATCC 14028* | sewage, Qingdao, China | 85 |
| NC_031065.1 | Salmonella phage vB_SnwM_CGG4-1 | *Salmonella enterica* subsp. *enterica* serovar Newport | sewage, Guelph Wastewater Treatment Plant, Canada | 86 |
| NC_024121.1 | Serratia phage PS2 | *S. marcescens S2* | fishpond water | 87 |
| NC_025829.1 | Shigella phage pSs-1 | *Shigella sonnei* ATCC 25931 | water, Hongjecheon stream, South Korea | 88 |

| | | | | |
|---|---|---|---|---|
| MF327006.1 | Shigella phage Sf20 | *Shigella boydii; Shigella flexneri* | water, Red Cedar River, USA | 89 |
| NC_042077.1 | Shigella phage Sf21 | *Shigella flexneri; Shigella sonnei; E.coli* K12 | water, Red Cedar River, USA | 89 |
| NC_042039.1 | Shigella phage Sf22 | *Shigella flexneri, Shigella boydii, Shigella dysenteriae, Shigella sonnei* | biofilm, buoy in the middle of the Grand River, USA | 89 |
| MF158046.1 | Shigella phage Sf23 | *Shigella flexneri, Shigella boydii, Shigella dysenteriae* | biofilm, buoy in the middle of the Grand River, USA | 89 |
| NC_042078.1 | Shigella phage Sf24 | *Shigella flexneri; Shigella sonnei; E.coli* K12 | water, Red Cedar River, USA | 89 |
| MF327009.1 | Shigella phage Sf25 | *Shigella flexneri; Shigella sonnei; E.coli* K12, *Shigella dysenteriae* | water, Red Cedar River, USA | 89 |
| KX828711.1 | Shigella phage SH7 | *Shigella flexneri* | water, wadi of Chotrana, Tunisia | 90 |
| NC_031085.1 | Shigella phage SHBML-50-1 | *Shigella sonnei* | surface water, Maryland park, USA | 102 |
| NC_015457.1 | Shigella phage Shfl2 | *Shigella flexneri* 37 | sewage water, Brazil | 102 |
| NC_030953.1 | Shigella phage SHFML-11 | *Shigella sonnei* | therapeutic phage library, Eliava Institute of Bacteriophage, Georgia | 102 |
| NC_031011.1 | Shigella phage SHFML-26 | *Shigella sonnei* | therapeutic phage library, Eliava Institute of Bacteriophage, Georgia | 102 |
| NC_031090.1 | Shigella phage SHSML-52-1 | *Shigella sonnei* | surface water, Maryland park, USA | 102 |
| NC_014595.1 | Shigella phage SP18 | *Shigella sonnei* | water, Gap River, South Korea | 91 |
| NC_005083.2 | Vibrio phage KVP40 | *Vibrio parahaemolyticus* | polluted sea water, Urado Bay, Japan | 92,93 |
| NC_021529.2 | Vibrio phage nt-1 | *Vibrio natriegens* | saltmarsh mud, Gloucester County, USA | 99 |
| KT919972.1 | Vibrio phage phi-Grn1 | *Vibrio alginolyticus* | coastal seawater, Crete, Greece | 94 |
| KT919973.1 | Vibrio phage phi-ST2 | *Vibrio alginolyticus* | coastal seawater, Crete, Greece | 94 |
| MK568540.1 | Vibrio phage ValB1_HC | *Vibrio alginolyticus* | - | - |
| NC_028829.1 | Vibrio phage ValKK3 | *Vibrio alginolyticus* | marine sediment, Kota Kinabalu, Malaysia | 103 |
| KU160494.1 | Vibrio phage vB_VmeM-32 | *Vibrio metschnikovii* DSM 29715 | sediment sample, lake Elmenteita, Kenya | 95 |
| NC_023568.1 | Vibrio phage VH7D | *Vibrio harveyi* | seawater, Xiamen abalone farm, China | 96 |
| JN849462.1 | Vibriophage phi-pp2 | *Vibrio parahaemolyticus* | water, aquaculture waterways around southern Taiwan, Taiwan | 97 |
| LR215722.1 | Yersinia phage fPS-2 | *Y. pseudotuberculosis* O:1a | pig stools, pig farms Finland | 98 |

| | | | | |
|---|---|---|---|---|
| NC_027353.1 | Yersinia phage phiD1 | *Yersinia pestis* | - | - |
| NC_027404.1 | Yersinia phage PST | *Yersinia pseudotuberculosis* | - | - |

"-" - no data was found

"*" - data were obtained from literature and genome annotation, BioSample database

## References

1. Petrov, V. M., Nolan, J. M., Bertrand, C., Levy, D., Desplats, C., Krisch, H. M., and Karam, J. D. (2006). Plasticity of the gene functions for DNA replication in the T4-like phages. *Journal of molecular biology* 361, 46–68. doi: 10.1016/j.jmb.2006.05.071

2. Coffi, H. (1995). *Lysotypie des Acinetobacter*. Master's Thesis. Laval University, Quebec, Canada

3. Petrov, V. M., Ratnayaka, S., Nolan, J. M., Miller, E. S., and Karam, J. D. (2010). Genomes of the T4-related bacteriophages as windows on microbial genome evolution. *Virology journal* 7:292. doi: 10.1186/1743-422X-7-292

4. Jansen, M., Wahida, A., Latz, S., Krüttgen, A., Häfner, H., Buhl, E. M., Ritter, K., and Horz, H. P. (2018). Enhanced antibacterial effect of the novel T4-like bacteriophage KARL-1 in combination with antibiotics against multi-drug resistant *Acinetobacter baumannii*. *Scientific reports* 8:14140. doi: 10.1038/s41598-018-32344-y

5. Pulkkinen, E., Wicklund, A., Oduor, J., Skurnik, M., and Kiljunen, S. (2019). Characterization of vB_ApiM_fHyAci03, a novel lytic bacteriophage that infects clinical *Acinetobacter* strains. *Archives of virology* 164, 2197–2199. doi: 10.1007/s00705-019-04284-z

6. Jin, J., Li, Z. J., Wang, S. W., Wang, S. M., Chen, S. J., Huang, D. H., Zhang, G., Li, Y. H., Wang, X. T., Wang, J., and Zhao, G. Q. (2014). Genome organisation of the *Acinetobacter* lytic phage ZZ1 and comparison with other T4-like Acinetobacter phages. *BMC genomics* 15:793. doi.: 10.1186/1471-2164-15-793

7. Jin, J., Li, Z. J., Wang, S. W., Wang, S. M., Huang, D. H., Li, Y. H., Ma, Y. Y., Wang, J., Liu, F., Chen, X. D., Li, G. X., Wang, X. T., Wang, Z. Q., and Zhao, G. Q. (2012). Isolation and characterization of ZZ1, a novel lytic phage that infects *Acinetobacter baumannii* clinical isolates. *BMC microbiology* 12:156. doi: 10.1186/1471-2180-12-156

8. Popoff, M. 1971. Étude sur les *Aeromonas salmonicida*, II. Caractérisation des bactériophages actifs sur les «*Aeromonas salmonicida*» et lysotypie. *Annales de Recherche Vétérinaires* 2, 33–45

9. Ackermann, H. W., Dauguet, C., Paterson, W. D., et al. (1985). *Aeromonas* bacteriophages: reexamination and classification. *Ann Inst Pasteur Vir*.136, 175–199

10. Vincent, A. T., Paquet, V. E., Bernatchez, A., Tremblay, D. M., Moineau, S., and Charette, S. J. (2017). Characterization and diversity of phages infecting *Aeromonas salmonicida* subsp. *salmonicida*. *Scientific reports* 7:7054. doi: 10.1038/s41598-017-07401-7

11. Chow, M. S., and Rouf, M. A. (1983). Isolation and Partial Characterization of Two *Aeromonas hydrophila* Bacteriophages. *Applied and environmental microbiology* 45, 1670–1676. doi: 10.1128/AEM.45.5.1670-1676.1983

12. Ackermann, H. W., and Krisch, H. M. (1997). A catalogue of T4-type bacteriophages. *Archives of virology* 142, 2329–2345. doi: 10.1007/s007050050246

13. Tétart, F., Desplats, C., Kutateladze, M., Monod, C., Ackermann, H. W., and Krisch, H. M. (2001). Phylogeny of the major head and tail genes of the wide-ranging T4-type bacteriophages. *Journal of bacteriology* 183, 358–366. doi: 10.1128/JB.183.1.358-366.2001

14. Chen, L., Yuan, S., Liu, Q., Mai, G., Yang, J., Deng, D., Zhang, B., Liu, C., and Ma, Y. (2018). In Vitro Design and Evaluation of Phage Cocktails Against *Aeromonas salmonicida*. *Frontiers in microbiology* 9:1476. doi:10.3389/fmicb.2018.01476

15. Shen, C. J., Liu, Y. J., and Lu, C. P. (2012). Complete Genome Sequence of *Aeromonas hydrophila* Phage CC2. *Journal of virology* 86:10900. doi: 10.1128/JVI.01882-12

16. Kim, J. H., Son, J. S., Choi, Y. J., Choresca, C. H., Jr, Shin, S. P., Han, J. E., Jun, J. W., and Park, S. C. (2012). Complete genome sequence and characterization of a broad-host range T4-like bacteriophage phiAS5 infecting *Aeromonas salmonicida* subsp. *salmonicida*. *Veterinary microbiology* 157, 164–171. doi: 10.1016/j.vetmic.2011.12.016

17. Russell, R. L. (1967). *Speciation among the T-even bacteriophages*. Ph.D. thesis, California Institute of Technology, Pasadena, CA.

18. McDermott, J. R., Shao, Q., O'Leary, C., Kongari, R., and Liu, M. (2019). Complete Genome Sequence of *Citrobacter freundii* Myophage Maroon. *Microbiology resource announcements* 8:e01145-19. doi: 10.1128/MRA.01145-19

19. LeSage, K. C., Hargrove, E. C., Cahill, J. L., Rasche, E. S., and Kuty Everett, G. F. (2015). Complete Genome Sequence of *Citrobacter freundii* Myophage Merlin. *Genome announcements* 3:e01133-15. doi: 10.1128/genomeA.01133-15

20. Hwang, K., Luna, A. J., Hernandez, A. C., and Kuty Everett, G. F. (2015). Complete Genome Sequence of *Citrobacter freundii* Myophage Miller. *Genome announcements* 3:e01425-14. doi: 10.1128/genomeA.01425-14

21. Edwards, G. B., Luna, A. J., Hernandez, A. C., and Kuty Everett, G. F. (2015). Complete Genome Sequence of *Citrobacter freundii* Myophage Moon. *Genome announcements* 3:e01427-14. doi: 10.1128/genomeA.01427-14

22. Oliveira, H., Pinto, G., Oliveira, A., Oliveira, C., Faustino, M. A., Briers, Y., Domingues, L., and Azeredo, J. (2016). Characterization and genome sequencing of a *Citrobacter freundii* phage CfP1 harboring a lysin active against multidrug-resistant isolates. *Applied microbiology and biotechnology* 100, 10543–10553. doi: 10.1007/s00253-016-7858-0

23. Madurkay, M., Kadličeková, V., Turňa, J., and Drahovska, H. (2019). Bacteriophage application for control of *Cronobacter* in liquid media and in biofilms. *Journal of Food & Nutrition Research* 58, 85-91

24. Abbasifar, R., Kropinski, A. M., Sabour, P. M., Ackermann, H. W., Lingohr, E. J., and Griffiths, M. W. (2012). Complete genome sequence of *Cronobacter sakazakii* bacteriophage vB_CsaM_GAP161. *Journal of virology*, 86, 13806–13807. doi: 10.1128/JVI.02546-12

25. Endersen, L., Buttimer, C., Nevin, E., Coffey, A., Neve, H., Oliveira, H., Lavigne, R., and O'Mahony, J. (2017). Investigating the biocontrol and anti-biofilm potential of a three phage cocktail against *Cronobacter sakazakii* in different brands of infant formula. *International journal of food microbiology* 253, 1–11. doi: 10.1016/j.ijfoodmicro.2017.04.009

26. Manohar, P., Tamhankar, A. J., Lundborg, C. S., and Nachimuthu, R. (2019). Therapeutic Characterization and Efficacy of Bacteriophage Cocktails Infecting *Escherichia coli*, *Klebsiella pneumoniae*, and *Enterobacter* Species. *Frontiers in microbiology* 10:574. doi: 10.3389/fmicb.2019.00574

27. Zhao, J., Zhang, Z., Tian, C., Chen, X., Hu, L., Wei, X., Li, H., Lin, W., Jiang, A., Feng, R., Yuan, J., Yin, Z., and Zhao, X. (2019). Characterizing the Biology of Lytic Bacteriophage vB_EaeM_φEap-3 Infecting Multidrug-Resistant *Enterobacter aerogenes*. *Frontiers in microbiology* 10:420. doi: 10.3389/fmicb.2019.00420

28. Ronner, A. B., and Cliver, D. O. (1990). Isolation and Characterization of a Coliphage Specific for *Escherichia coli* 0157:H7. *Journal of food protection* 53, 944–947. doi: 10.4315/0362-028X-53.11.944

29. Zhang, C., Li, W., Liu, W., Zou, L., Yan, C., Lu, K., and Ren, H. (2013). T4-like phage Bp7, a potential antimicrobial agent for controlling drug-resistant *Escherichia coli* in chickens. *Applied and environmental microbiology,* 79, 5559–5565. doi: 10.1128/AEM.01505-13

30. Merabishvili, M., De Vos, D., Verbeken, G., Kropinski, A. M., Vandenheuvel, D., Lavigne, R., Wattiau, P., Mast, J., Ragimbeau, C., Mossong, J., Scheres, J., Chanishvili, N., Vaneechoutte, M., and Pirnay, J. P. (2012). Selection and characterization of a candidate therapeutic bacteriophage that lyses the *Escherichia coli* O104:H4 strain from the 2011 outbreak in Germany. *PloS one* 7:e52709. doi: 10.1371/journal.pone.0052709

31. Tang, F., Li, Y., Zhang, W., and Lu, C. (2012). Complete genome sequence of T4-Like *Escherichia coli* bacteriophage HX01. *Journal of virology* 86:13871. doi: 10.1128/JVI.02698-12

32. Jiang, X., Jiang, H., Li, C., Wang, S., Mi, Z., An, X., Chen, J., and Tong, Y. (2011). Sequence characteristics of T4-like bacteriophage IME08 benome termini revealed by high throughput sequencing. *Virology journal* 8:194. doi: 10.1186/1743-422X-8-194

33. Denou, E., Bruttin, A., Barretto, C., Ngom-Bru, C., Brüssow, H., and Zuber, S. (2009). T4 phages against *Escherichia coli* diarrhea: potential and problems. *Virology* 388, 21–30. doi: 10.1016/j.virol.2009.03.009

34. Arbiol, C., Comeau, A. M., Kutateladze, M., Adamia, R., and Krisch, H. M. (2010). Mobile regulatory cassettes mediate modular shuffling in T4-type phage genomes. *Genome biology and evolution* 2, 140–152. doi: 10.1093/gbe/evq006

35. Xu, J., Chen, M., He, L., Zhang, S., Ding, T., Yao, H., Lu, C., and Zhang, W. (2016). Isolation and characterization of a T4-like phage with a relatively wide host range within *Escherichia coli*. *Journal of basic microbiology* 56, 405–421. doi: 10.1002/jobm.201500440

36. Abedon, S. T. (2000). The murky origin of Snow White and her T-even dwarfs. *Genetics*, 155, 481–486

37. Anderson, T. F. (1944). Virus reactions inside of bacterial host cells. *J. Bact*, 47:113

38. Bryson, A. L., Hwang, Y., Sherrill-Mix, S., Wu, G. D., Lewis, J. D., Black, L., Clark, T. A., and Bushman, F. D. (2015). Covalent Modification of Bacteriophage T4 DNA Inhibits CRISPR-Cas9. *mBio* 6:e00648. doi: 10.1128/mBio.00648-15

39. Chibeu, A., Lingohr, E. J., Masson, L., Manges, A., Harel, J., Ackermann, H. W., Kropinski, A. M., and Boerlin, P. (2012). Bacteriophages with the ability to degrade uropathogenic *Escherichia coli* biofilms. *Viruses* 4, 471–487. doi: 10.3390/v4040471

40. Li, P., Lin, H., Mi, Z., Xing, S., Tong, Y., and Wang, J. (2019). Screening of Polyvalent Phage-Resistant *Escherichia coli* Strains Based on Phage Receptor Analysis. *Frontiers in microbiology* 10:850. doi: 10.3389/fmicb.2019.00850

41. Kaliniene, L., Klausa, V., and Truncaite, L. (2010). Low-temperature T4-like coliphages vB_EcoM-VR5, vB_EcoM-VR7 and vB_EcoM-VR20. *Archives of virology* 155, 871–880. doi: 10.1007/s00705-010-0656-6

42. Kaliniene, L., Zajančkauskaitė, A., Šimoliūnas, E., Truncaitė, L., and Meškys, R. (2015). Low-temperature bacterial viruses VR - a small but diverse group of *E. coli* phages. *Archives of virology* 160, 1367–1370. doi: 10.1007/s00705-015-2388-0

43. Cowley, L. A., Beckett, S. J., Chase-Topping, M., Perry, N., Dallman, T. J., Gally, D. L., and Jenkins, C. (2015). Analysis of whole genome sequencing for the *Escherichia coli* O157:H7 typing phages. *BMC genomics* 16:271. doi: 10.1186/s12864-015-1470-z

44. Dalmasso, M., Strain, R., Neve, H., Franz, C. M., Cousin, F. J., Ross, R. P., and Hill, C. (2016). Three New *Escherichia coli* Phages from the Human Gut Show Promising Potential for Phage Therapy. *PloS one* 11:e0156773. doi: 10.1371/journal.pone.0156773

45. Green, S. I., Kaelber, J. T., Ma, L., Trautner, B. W., Ramig, R. F., and Maresso, A. W. (2017). Bacteriophages from ExPEC Reservoirs Kill Pandemic Multidrug-Resistant Strains of Clonal Group ST131 in Animal Models of Bacteremia. *Scientific reports* 7:46151. doi: 10.1038/srep46151

46. Korf, I., Meier-Kolthoff, J. P., Adriaenssens, E. M., Kropinski, A. M., Nimtz, M., Rohde, M., van Raaij, M. J., and Wittmann, J. (2019). Still Something to Discover: Novel Insights into *Escherichia coli* Phage Diversity and Taxonomy. *Viruses* 11:454. doi: 10.3390/v11050454

47. Coffey, B., Ross, R. P., O'Flynn, G., O'Sullivan, O., Casey, A., Callanan, M., Coffey, A., and McAuliffe, O. (2014). Complete Genome Sequence of vB_EcoM_112, a T-Even-Type Bacteriophage Specific for *Escherichia coli* O157:H7. *Genome announcements* 2:e00393-14. doi: 10.1128/genomeA.00393-14

48. O'Flynn, G., Ross, R. P., Fitzgerald, G. F., and Coffey, A. (2004). Evaluation of a cocktail of three bacteriophages for biocontrol of *Escherichia coli* O157:H7. *Applied and environmental microbiology* 70, 3417–3424. doi: 10.1128/AEM.70.6.3417-3424.2004

49. Aleshkin, A. V., Rubalskii, E. O., Volozhantsev, N. V., Verevkin, V. V., Svetoch, E. A., Kiseleva, I. A., Bochkareva, S. S., Borisova, O. Y., Popova, A. V., Bogun, A. G., and Afanas'ev, S. S. (2015). A small-scale experiment of using phage-based probiotic dietary supplement for prevention of *E. coli* traveler's diarrhea. *Bacteriophage* 5:e1074329. doi: 10.1080/21597081.2015.1074329

50. Aleshkin, A. V., Volozhantsev, N. V., Svetoch, E. A., Kiseleva, I. A., Rubal'sky, E. O., Afanas’ev, S. S., et al. (2016). Bacteriophages as probiotics: phage-based probiotic dietary supplement in prophylaxis against foodborne infections. *Infekc. Bolezni* 14, 31-40

51. Carter, C. D., Parks, A., Abuladze, T., Li, M., Woolston, J., Magnone, J., Senecal, A., Kropinski, A. M., and Sulakvelidze, A. (2012). Bacteriophage cocktail significantly reduces *Escherichia coli* O157: H7 contamination of lettuce and beef, but does not protect against recontamination. *Bacteriophage* 2, 178–185. doi: 10.4161/bact.22825

52. Lee, H., Ku, H. J., Lee, D. H., Kim, Y. T., Shin, H., Ryu, S., and Lee, J. H. (2016). Characterization and Genomic Study of the Novel Bacteriophage HY01 Infecting Both *Escherichia coli* O157:H7 and Shigella flexneri: Potential as a Biocontrol Agent in Food. *PloS one*, 11:e0168985. doi: 10.1371/journal.pone.0168985
53. Pham-Khanh, N. H., Sunahara, H., Yamadeya, H., Sakai, M., Nakayama, T., Yamamoto, H., Truong Thi Bich, V., Miyanaga, K., and Kamei, K. (2019). Isolation, Characterisation and Complete Genome Sequence of a *Tequatrovirus* Phage, Escherichia phage KIT03, Which Simultaneously Infects *Escherichia coli* O157:H7 and *Salmonella enterica*. *Current microbiology* 76, 1130–1137. doi: 10.1007/s00284-019-01738-0
54. Kushkina, A. I., Tovkach, F. I., Comeau, A. M., Kostetskii, I. E., Lisovski, I., Ostapchuk, A. M., Voychuk, S. I., Gorb, T. I., and Romaniuk, L. V. (2013). Complete Genome Sequence of Escherichia Phage Lw1, a New Member of the RB43 Group of Pseudo T-Even Bacteriophages. *Genome announcements* 1:e00743-13. doi: 10.1128/genomeA.00743-13
55. Howard-Varona, C., Vik, D. R., Solonenko, N. E., Gazitua, M. C., Hobbs, Z., Honaker, R. W., Kinkhabwala, A. A., and Sullivan, M. B. (2018). Whole-Genome Sequences of Phages p000v and p000y, Which Infect the Bacterial Pathogen Shiga-Toxigenic *Escherichia coli*. *Microbiology resource announcements* 7:e01400-18. doi:10.1128/MRA.01400-18
56. Morita, M., Tanji, Y., Mizoguchi, K., Akitsu, T., Kijima, N., and Unno, H. (2002). Characterization of a virulent bacteriophage specific for *Escherichia coli* O157:H7 and analysis of its cellular receptor and two tail fiber genes. *FEMS microbiology letters* 211, 77–83. doi: 10.1111/j.1574-6968.2002.tb11206.x
57. Zaman, S. F. J. (2014*). Isolation and Characterization of Bacteriophage from Raw Sewage Specific for Escherichia Coli 0157: H7*. Ph.D. thesis, Universiti Sains Malaysia, Penang, Malaysia
58. Smith, R., O'Hara, M., Hobman, J. L., and Millard, A. D. (2015). Draft Genome Sequences of 14 *Escherichia coli* Phages Isolated from Cattle Slurry. *Genome announcements* 3:e01364-15. doi: 10.1128/genomeA.01364-15
59. Liu, H., Liu, X., and Li, J. (2017). Complete genome of a novel virulent phage ST0 lysing *Escherichia coli* H8. *Standards in genomic sciences* 12:85. doi: 10.1186/s40793-017-0304-9
60. Lopez, M. E., Batalha, L. S., Vidigal, P. M., Albino, L. A., Boggione, D. M., Gontijo, M. T., Bazzolli, D. M., and Mendonca, R. C. (2016). Genome Sequence of the Enterohemorrhagic *Escherichia coli* Bacteriophage UFV-AREG1. *Genome announcements*, 4:e00412-16. doi: 10.1128/genomeA.00412-16

61. Barros, J., Melo, L., Poeta, P., Igrejas, G., Ferraz, M. P., Azeredo, J., and Monteiro, F. J. (2019). Lytic bacteriophages against multidrug-resistant *Staphylococcus aureus*, *Enterococcus faecalis* and *Escherichia coli* isolates from orthopaedic implant-associated infections. *International journal of antimicrobial agents* 54, 329–337. doi: 10.1016/j.ijantimicag.2019.06.007
62. Zhou, Y., Bao, H., Zhang, H., and Wang, R. (2015). Isolation and Characterization of Lytic Phage vB_EcoM_JS09 against Clinically Isolated Antibiotic-Resistant Avian Pathogenic *Escherichia coli* and Enterotoxigenic *Escherichia coli*. *Intervirology* 58, 218–231. doi: 10.1159/000437426
63. Tsonos, J., Oosterik, L. H., Tuntufye, H. N., Klumpp, J., Butaye, P., De Greve, H., Hernalsteens, J. P., Lavigne, R., and Goddeeris, B. M. (2014). A cocktail of in vitro efficient phages is not a guarantee for in vivo therapeutic results against avian colibacillosis. *Veterinary microbiology* 171, 470–479. doi: 10.1016/j.vetmic.2013.10.021
64. Kiljunen, S., Wicklund, A., and Skurnik, M. (2018). Complete Genome Sequences of Two Escherichia Phages Isolated from Wastewater in Finland. *Genome announcements* 6:e00401-18. doi: 10.1128/genomeA.00401-18
65. Liao, Y. T., Zhang, Y., Salvador, A., and Wu, V. (2019). Genome Sequence of a T4-like Phage, Escherichia Phage vB_EcoM-Sa45lw, Infecting Shiga Toxin-Producing *Escherichia coli* Strains. *Microbiology resource announcements* 8:e00804-19. doi: 10.1128/MRA.00804-19
66. Duarte, V. S., Dias, R. S., Kropinski, A. M., Vidigal, P. M., Sousa, F. O., Xavier, A. S., Silva, C. C., and de Paula, S. O. (2016). Complete Genome Sequence of vB_EcoM-UFV13, a New Bacteriophage Able To Disrupt *Trueperella pyogenes* Biofilm. *Genome announcements*, 4:e01292-16. doi: 10.1128/genomeA.01292-16
67. Chen, M., Zhang, L., Abdelgader, S. A., Yu, L., Xu, J., Yao, H., Lu, C., and Zhang, W. (2017). Alterations in gp37 Expand the Host Range of a T4-Like Phage. *Applied and environmental microbiology*, 83:e01576-17. doi: 10.1128/AEM.01576-17
68. Ahmed, R., Bopp, C., Borczyk, A., and Kasatiya, S. (1987). Phage-typing scheme for *Escherichia coli* O157:H7. *The Journal of infectious diseases* 155, 806–809. doi: 10.1093/infdis/155.4.806
69. Kropinski, A. M., Lingohr, E. J., Moyles, D. M., Chibeu, A., Mazzocco, A., Franklin, K., Villegas, A., Ahmed, R., She, Y. M., & Johnson, R. P. (2012). *Escherichia coli* O157:H7 typing phage V7 is a T4-like virus. *Journal of virology*, 86:10246. doi: 10.1128/JVI.01642-12
70. Michniewski, S., Redgwell, T., Grigonyte, A., Rihtman, B., Aguilo-Ferretjans, M., Christie-Oleza, J., Jameson, E., Scanlan, D. J., and Millard, A. D. (2019).

Riding the wave of genomics to investigate aquatic coliphage diversity and activity. *Environmental microbiology* 21, 2112–2128. doi: 10.1111/1462-2920.14590

71. Kim, Y., Bang, I., Yeon, Y. E., Park, J. Y., Han, B. K., Kim, H., and Kim, D. (2018). Draft genome sequence of lytic bacteriophage KP1 infecting bacterial pathogen *Klebsiella pneumoniae*. *The Microbiological Society of Korea* 54, 152-154.

72. Harb, L., Boeckman, J., Newkirk, H., Liu, M., Gill, J. J., and Ramsey, J. (2019). Complete Genome Sequence of the Novel *Klebsiella pneumoniae* Phage Marfa. *Microbiology resource announcements* 8:e00748-19. doi: 10.1128/MRA.00748-19

73. Provasek, V. E., Lessor, L. E., Cahill, J. L., Rasche, E. S., and Kuty Everett, G. F. (2015). Complete Genome Sequence of Carbapenemase-Producing *Klebsiella pneumoniae* Myophage Matisse. *Genome announcements* 3:e01136-15. doi: 10.1128/genomeA.01136-15

74. Boeckman, J. X., Lessor, L., Gill, J. J., and Liu, M. (2019). Complete Genome Sequence of *Klebsiella pneumoniae* Myophage Mineola. *Microbiology resource announcements*, 8:e00257-19. doi: 10.1128/MRA.00257-19

75. Mijalis, E. M., Lessor, L. E., Cahill, J. L., Rasche, E. S., and Kuty Everett, G. F. (2015). Complete Genome Sequence of *Klebsiella pneumoniae* Carbapenemase-Producing *K. pneumoniae* Myophage Miro. *Genome announcements*, 3:e01137-15. doi: 10.1128/genomeA.01137-15

76. Park, E. A., Kim, Y. T., Cho, J. H., Ryu, S., and Lee, J. H. (2017). Characterization and genome analysis of novel bacteriophages infecting the opportunistic human pathogens *Klebsiella oxytoca* and *K. pneumoniae*. *Archives of virology*, 162, 1129–1139. doi: 10.1007/s00705-016-3202-3

77. Ciacci, N., D'Andrea, M. M., Marmo, P., Demattè, E., Amisano, F., Di Pilato, V., Fraziano, M., Lupetti, P., Rossolini, G. M., and Thaller, M. C. (2018). Characterization of vB_Kpn_F48, a Newly Discovered Lytic Bacteriophage for *Klebsiella pneumoniae* of Sequence Type 101. *Viruses* 10:482. doi: 10.3390/v10090482

78. Komisarova, E. V., Kislichkina, A. A., Krasilnikova, V. M., Bogun, A. G., Fursova, N. K., and Volozhantsev, N. V. (2017). Complete Nucleotide Sequence of *Klebsiella pneumoniae* Bacteriophage vB_KpnM_KpV477. *Genome announcements*, 5:e00694-17. doi: 10.1128/genomeA.00694-17

79. Oliveira, H., Pinto, G., Oliveira, A., Noben, J. P., Hendrix, H., Lavigne, R., Łobocka, M., Kropinski, A. M., and Azeredo, J. (2017). Characterization and genomic analyses of two newly isolated *Morganella* phages define distant

members among *Tevenvirinae* and *Autographivirinae* subfamilies. *Scientific reports* 7:46157. doi: 10.1038/srep46157

80. Lim, J. A., Lee, D. H., and Heu, S. (2015). Isolation and Genomic Characterization of the T4-Like Bacteriophage PM2 Infecting *Pectobacterium carotovorum* subsp. *carotovorum. The plant pathology journal* 31, 83–89. doi: 10.5423/PPJ.NT.09.2014.0099

81. Gogokhia, L., Buhrke, K., Bell, R., Hoffman, B., Brown, D. G., Hanke-Gogokhia, C., Ajami, N. J., Wong, M. C., Ghazaryan, A., Valentine, J. F., Porter, N., Martens, E., O'Connell, R., Jacob, V., Scherl, E., Crawford, C., Stephens, W. Z., Casjens, S. R., Longman, R. S., and Round, J. L. (2019). Expansion of Bacteriophages Is Linked to Aggravated Intestinal Inflammation and Colitis. *Cell host & microbe* 25, 285.e8–299.e8. doi: 10.1016/j.chom.2019.01.008

82. Zhang, K., Xie, Y., O'Leary, C. J., Liu, M., and Gill, J. J. (2019). Complete Genome Sequence of *Salmonella enterica* Serovar Newport Myophage Melville. *Microbiology resource announcements* 8:e00255-19. doi: 10.1128/MRA.00255-19

83. Marti, R., Zurfluh, K., Hagens, S., Pianezzi, J., Klumpp, J., and Loessner, M. J. (2013). Long tail fibres of the novel broad-host-range T-even bacteriophage S16 specifically recognize Salmonella OmpC. *Molecular microbiology* 87, 818–834. doi: 10.1111/mmi.12134

84. Woolston, J., Parks, A. R., Abuladze, T., Anderson, B., Li, M., Carter, C., Hanna, L. F., Heyse, S., Charbonneau, D., and Sulakvelidze, A. (2013). Bacteriophages lytic for *Salmonella* rapidly reduce *Salmonella* contamination on glass and stainless steel surfaces. *Bacteriophage* 3:e25697. doi: 10.4161/bact.25697

85. Li, M., Li, M., Lin, H., Wang, J., Jin, Y., and Han, F. (2016). Characterization of the novel T4-like *Salmonella enterica* bacteriophage STP4-a and its endolysin. *Archives of virology*, 161, 377–384. doi: 10.1007/s00705-015-2647-0

86. El-Dougdoug, N. K., Cucic, S., Abdelhamid, A. G., Brovko, L., Kropinski, A. M., Griffiths, M. W., and Anany, H. (2019). Control of *Salmonella* Newport on cherry tomato using a cocktail of lytic bacteriophages. *International journal of food microbiology* 293, 60–71. doi: 10.1016/j.ijfoodmicro.2019.01.003

87. Teng, T., Zhang, G., Fan, X., Zhang, Z., Zhang, L., Wu, D., Chen, S., Li, Y., and Jin, J. (2018). Complete genome sequence analysis of PS2, a novel T4-like bacteriophage that infects *Serratia marcescens* clinical isolates. *Archives of virology* 163, 1997–2000. doi: 10.1007/s00705-018-3803-0

88. Jun, J. W., Giri, S. S., Kim, H. J., Yun, S. K., Chi, C., Chai, J. Y., Lee, B. C., and Park, S. C. (2016). Bacteriophage application to control the contaminated water with *Shigella*. *Scientific reports* 6:22636. doi: 10.1038/srep22636

89. Doore, S. M., Schrad, J. R., Dean, W. F., Dover, J. A., and Parent, K. N. (2018). *Shigella* Phages Isolated during a Dysentery Outbreak Reveal Uncommon Structures and Broad Species Diversity. *Journal of virology* 92:e02117-17. doi: 10.1128/JVI.02117-17

90. Hamdi, S., Rousseau, G. M., Labrie, S. J., Tremblay, D. M., Kourda, R. S., Ben Slama, K., and Moineau, S. (2017). Characterization of two polyvalent phages infecting *Enterobacteriaceae*. *Scientific reports*, 7:40349. doi: 10.1038/srep40349

91. Kim, K. H., Chang, H. W., Nam, Y. D., Roh, S. W., and Bae, J. W. (2010). Phenotypic characterization and genomic analysis of the *Shigella sonnei* bacteriophage SP18. *Journal of microbiology (Seoul, Korea)* 48, 213–222. doi: 10.1007/s12275-010-0055-4

92. Matsuzaki, S., Tanaka, S., Koga, T., and Kawata, T. (1992). A broad-host-range vibriophage, KVP40, isolated from sea water. *Microbiology and immunology* 36, 93–97. doi: 10.1111/j.1348-0421.1992.tb01645.x

93. Miller, E. S., Heidelberg, J. F., Eisen, J. A., Nelson, W. C., Durkin, A. S., Ciecko, A., Feldblyum, T. V., White, O., Paulsen, I. T., Nierman, W. C., Lee, J., Szczypinski, B., and Fraser, C. M. (2003). Complete genome sequence of the broad-host-range vibriophage KVP40: comparative genomics of a T4-related bacteriophage. *Journal of bacteriology* 185, 5220–5233. doi: 10.1128/jb.185.17.5220-5233.2003

94. Skliros, D., Kalatzis, P. G., Katharios, P., & Flemetakis, E. (2016). Comparative Functional Genomic Analysis of Two *Vibrio* Phages Reveals Complex Metabolic Interactions with the Host Cell. *Frontiers in microbiology* 7:1807. doi: 10.3389/fmicb.2016.01807

95. Akhwale, J. K., Rohde, M., Rohde, C., Bunk, B., Spröer, C., Boga, H. I., Klenk, H. P., and Wittmann, J. (2019). Isolation, characterization and analysis of bacteriophages from the haloalkaline lake Elmenteita, Kenya. *PloS one* 14:e0215734. doi: 10.1371/journal.pone.0215734

96. Luo, Z. H., Yu, Y. P., Jost, G., Xu, W., and Huang, X. L. (2015). Complete genome sequence of a giant Vibrio bacteriophage VH7D. *Marine genomics* 24, 293–295. doi: 10.1016/j.margen.2015.10.005

97. Lin, Y. R., and Lin, C. S. (2012). Genome-wide characterization of Vibrio phage φpp2 with unique arrangements of the mob-like genes. *BMC genomics* 13:224. doi: 10.1186/1471-2164-13-224

98. Salem, M., Virtanen, S., Korkeala, H., and Skurnik, M. (2015). Isolation and characterization of *Yersinia*-specific bacteriophages from pig stools in Finland. *Journal of applied microbiology* 118, 599–608. doi: 10.1111/jam.12722

99. Comeau, A. M., Arbiol, C., and Krisch, H. M. (2014). Composite conserved promoter-terminator motifs (PeSLs) that mediate modular shuffling in the diverse T4-like myoviruses. *Genome biology and evolution* 6, 1611–1619. doi: 10.1093/gbe/evu129

100. Son, H. M., Duc, H. M., Masuda, Y., Honjoh, K. I., and Miyamoto, T. (2018). Application of bacteriophages in simultaneously controlling *Escherichia coli* O157:H7 and extended-spectrum beta-lactamase producing *Escherichia coli*. *Applied microbiology and biotechnology* 102, 10259–10271. doi: 10.1007/s00253-018-9399-1

101. da Silva Duarte, V., Dias, R. S., Kropinski, A. M., Campanaro, S., Treu, L., Siqueira, C., Vieira, M. S., da Silva Paes, I., Santana, G. R., Martins, F., Crispim, J. S., da Silva Xavier, A., Ferro, C. G., Vidigal, P., da Silva, C. C., and de Paula, S. O. (2018). Genomic analysis and immune response in a murine mastitis model of vB_EcoM-UFV13, a potential biocontrol agent for use in dairy cows. *Scientific reports* 8:6845. doi: 10.1038/s41598-018-24896-w

102. Subramanian, S., Parent, K. N., and Doore, S. M. (2020). Ecology, Structure, and Evolution of *Shigella* Phages. *Annual Review of Virology* 7:1. doi:10.1146/annurev-virology-010320-052547

103. Lal, T. M., Sano, M., Hatai, K., & Ransangan, J. (2016). Complete genome sequence of a giant Vibrio phage ValKK3 infecting *Vibrio alginolyticus*. *Genomics data* 8, 37–38. doi: 10.1016/j.gdata.2016.03.002
