# Supplementary material for: Influence of Non-canonical DNA Bases on the Genomic Diversity of Tevenvirinae
Source: Front Microbiol. 2021 Apr 6;12:632686. doi: 10.3389/fmicb.2021.632686 (PMC8056088; doi:10.3389/fmicb.2021.632686)
Supplement: Supplementary Table 4 — GenBank accession numbers and IGM/VR Scaffolds IDs of genomes added for Additional Analysis. [file Table_4.PDF]

Accession number/Scaffold ID	Phage
MT385367.1	Acinetobacter_phage_Abraxas
NC_049445.1	Acinetobacter_phage_AbTZA1
NC_049491.1	Acinetobacter_phage_Apostate
NC_049490.1	Acinetobacter_phage_Berthold
MK240351.1	Acinetobacter_phage_Henu6
NC_049492.1	Acinetobacter_phage_Kimel
NC_049479.1	Acinetobacter_phage_Konradin
NC_049493.1	Acinetobacter_phage_Lazarus
MT741943.1	Acinetobacter_phage_Meroveus
MT409116.1	Acinetobacter_phage_Octan
MN662249.1	Acinetobacter_phage_Stupor
NC_049494.1	Acinetobacter_phage_vB_AbaM_PhT2
MT161459.1	Aeromonas_phage_Ahp1
MT713136.1	Aeromonas_phage_AP1
MN871440.1	Aeromonas_phage_AsFcp_1
MN871442.1	Aeromonas_phage_Aszh-1
MW272540.1	Aeromonas_phage_ZPAH1
MT334653.1	Buttiauxella_phage_vB_ButM_GuL6
NC_042067.1	Citrobacter_phage_CF1_ERZ-2017
MW239124.1	Citrobacter_phage_CkP1
MT176425.1	Citrobacter_phage_PhiZZ23
MT176424.1	Citrobacter_phage_PhiZZ6
MW021749.1	Citrobacter_phage_vB_Cfr_Xman
MG775043.2	Citrobacter_phage_vB_CroM_CrRp10
NC_028773.1	Cronobacter_phage_S13
MW021751.1	Cronobacter_phage_vB_CsaM_Cronuts
NC_048646.1	Cronobacter_phage_vB_CsaM_leE
MW021756.1	Cronobacter_phage_vB_CsaM_SemperBestia
NC_028683.1	Edwardsiella_phage_PEi20
AP014715.1	Edwardsiella_phage_PEi26
MT341500.1	Enterobacter_phage_EBPL
MN508623.1	Enterobacter_phage_EC-F1
MN508624.1	Enterobacter_phage_EC-F2
MN508621.1	Enterobacter_phage_EC-W1
MN508622.1	Enterobacter_phage_EC-W2
MN617835.1	Enterobacter_phage_prasa_myo
NC_048849.1	Enterobacter_phage_vB_EclM_CIP9
MN087708.1	Enterobacter_phage_vB_EhoM-IME523
LC589952.1	Enterobacter_phage_vB_EkoM5VN_DNA
NC_019503.1	Enterobacteria_phage_ime09
MH051913.1	Enterobacteria_phage_vB_EcoM_IME281
MH051917.1	Enterobacteria_phage_vB_EcoM_IME341
MT150133.1	Enterobacteria_phage_vB_EcoM_IME540
MT533174.1	Escherichia_phage_CJ20
MN850609.1	Escherichia_phage_dhaeg
MT611523.1	Escherichia_phage_DK-13
MN655998.1	Escherichia_phage_E26

MK886800.1	Escherichia_phage_EcNP1
MN508614.1	Escherichia_phage_ES12
MN508616.1	Escherichia_phage_ES19
MN508617.1	Escherichia_phage_ES21
MN508618.1	Escherichia_phage_ES26
MN864145.1	Escherichia_phage_F2
MT764206.1	Escherichia_phage_JEP6
MT764208.1	Escherichia_phage_JEP8
MT782071.1	Escherichia_phage_JN02
MN850574.1	Escherichia_phage_kaaroe
KX452694.1	Escherichia_phage_KNP1
MN850579.1	Escherichia_phage_mogra
MN850590.1	Escherichia_phage_moha
MN850651.1	Escherichia_phage_moskry
KR233165.1	Escherichia_phage_PEC04
MK524178.1	Escherichia_phage_PHB12
KY703222.1	Escherichia_phage_phiC120
LR597660.1	Escherichia_phage_T4_ev151
LR597657.1	Escherichia_phage_T4_ev240
MN895438.1	Escherichia_phage_teqdroes
MN895434.1	Escherichia_phage_teqhad
MN895435.1	Escherichia_phage_teqhal
MN895437.1	Escherichia_phage_teqskov
MN895436.1	Escherichia_phage_teqsoen
MT478991.1	Escherichia_phage_vB_EcoM_011D4
MT884007.2	Escherichia_phage_vb_EcoM_bov10K1
MT884006.2	Escherichia_phage_vb_EcoM_bov9_1
MT682712.1	Escherichia_phage_vB_EcoM_F1
MT682711.1	Escherichia_phage_vB_EcoM_FB
MT682710.1	Escherichia_phage_vB_EcoM_FT
MK327928.1	Escherichia_phage_vB_EcoM_G2133
MT179807.1	Escherichia_phage_vB_EcoM_IME537
MT682714.1	Escherichia_phage_vB_EcoM_Lutter
MH243438.1	Escherichia_phage_vB_EcoM_NBG1
MH243439.1	Escherichia_phage_vB_EcoM_NBG2
MT682713.1	Escherichia_phage_vB_EcoM_Ozark
MT682709.1	Escherichia_phage_vB_EcoM_SP1
MT968995.1	Escherichia_phage_vB_EcoM_WL-3
MW286157.1	Escherichia_phage_vB_EcoM-BECP11
MH837626.1	Escherichia_phage_vB_vPM_PD112
MT932213.1	Escherichia_phage_VEc74
KY290975.2	Escherichia_phage_YUEEL01
MN894885.1	Escherichia_virus_Ec_Makalu_001
MN709127.1	Escherichia_virus_Ec_Makalu_002
MN882349.1	Escherichia_virus_Ec_Makalu_003
MT446387.1	Escherichia_virus_TH09
MT446390.1	Escherichia_virus_TH12
MT446392.1	Escherichia_virus_TH15

MT446396.1	Escherichia_virus_TH22
MT446411.1	Escherichia_virus_TH40
MT446412.1	Escherichia_virus_TH41
MT446415.1	Escherichia_virus_TH44
MT446420.1	Escherichia_virus_TH54
MT446421.1	Escherichia_virus_TH55
MT446422.1	Escherichia_virus_TH57
MT446423.1	Escherichia_virus_TH58
MN434092.1	Klebsiella_phage_AmPh_EK29
CP062992.1	Klebsiella_phage_AShe-2020a
MN106245.1	Klebsiella_phage_EI
KT239446.1	Klebsiella_phage_JD18
MN101223.1	Klebsiella_phage_KOX10
NC_014036.1	Klebsiella_phage_KP15
MH729874.1	Klebsiella_phage_KP179
NC_020080.1	Klebsiella_phage_KP27
MN101230.1	Klebsiella_phage_KPN6
MT701588.1	Klebsiella_phage_Metamorpho
MT157285.1	Klebsiella_phage_P-KP2
MN395284.1	Klebsiella_phage_PhiKpNIH-6
NC_042138.1	Klebsiella_phage_PMBT1
MN013084.1	Klebsiella_phage_vB_KaeM_KaAlpha
MN781108.1	Klebsiella_phage_vB_Kpn_P545
LR746310.1	Klebsiella_phage_vB_KpnM_05F
LR877331.1	Klebsiella_phage_vB_KpnM_311F
MW021752.1	Klebsiella_phage_vB_KpnM_BovinicusUrsus
MN013081.1	Klebsiella_phage_vB_KpnM_Potts1
MN434095.1	Klebsiella_pneumoniae_phage_JIPh_Kp122
MN038175.1	Panteoa_phage_Phynn
MN994497.1	Phage_NBEco003
MW057858.1	Providencia_phage_PSTCR6
MW358927.1	Providencia_phage_PSTRCR_127
MN580668.1	Salmonella_phage_pSe_SNUABM_01
MW149274.1	Salmonella_phage_vB_SalM_ABTNLsp5
MW082584.1	Serratia_phage_4S
MN095771.1	Serratia_phage_Muldoon
MT176426.1	Serratia_phage_PhiZZ30
MT457552.1	Shewanella_phage_Thanatos-1
MT457553.1	Shewanella_phage_Thanatos-2
MK962750.1	Shigella_phage_CM8
MK962752.1	Shigella_phage_JK23
MK962753.1	Shigella_phage_JK32
MK962754.1	Shigella_phage_JK36
MK962755.1	Shigella_phage_JK38
MK962756.1	Shigella_phage_JK42
MK962757.1	Shigella_phage_JK45
KX452698.1	Shigella_phage_KNP5
MG589383.1	Shigella_phage_phi25-307

MW341595.1	Shigella_phage_Sfk20
LC465543.1	Shigella_phage_SfPhi01
NC_025437.1	Shigella_phage_Shf125875
MK639187.1	Shigella_phage_SSE1
MK295204.1	Shigella_phage_vB_SdyM_006
LR746311.1	Shigella_phage_vB_SsoM_113
LR877332.1	Shigella_phage_vB_SsoM_JK08
MN781580.1	Shigella_virus_KRT47
MK568540.1	Vibrio_phage_phiVa3
MT135025.1	Vibrio_phage_V07
MT135026.1	Vibrio_phage_V09
MT612988.1	Vibrio_phage_vB_ValM_R10Z
MT612989.1	Vibrio_phage_vB_ValM_R11Z
MN794232.1	Vibrio_phage_VH1_2019
LR215724.1	Yersinia_phage_fPS-65
LR215723.1	Yersinia_phage_fPS-90
MH809535.1	Yersinia_phage_PYPS2T
MN716856.1	Yersinia_phage_vB_YepM_ZN18
Ga0244934_100071	Ga0244934_100071
Ga0121214_100002	Ga0121214_100002
