# Supplementary material for: Influence of Non-canonical DNA Bases on the Genomic Diversity of Tevenvirinae
Source: Front Microbiol. 2021 Apr 6;12:632686. doi: 10.3389/fmicb.2021.632686 (PMC8056088; doi:10.3389/fmicb.2021.632686)
Supplement: Supplementary Data Sheet 1 — Clustering results (folders “first clusterisation,” “second clusterisation,” and “third clusterisation”): the number of pan-genome components in the individual genome (files “pan genome components of genomes”), the total number of all clusters and components of the pan-genome (files “pan genome components”), a list of clusters of the core genome (files “core list”), a list of clusters of the softcore genome (files “softcore list”). Clusters of the softcore genome (folders “softcore clusters”) are presented in the files which show regions in genomes, protein sequences, names of viruses which have these proteins A description of softcore clusters is also supplied (file “Softcore clusters description”). [file Data_Sheet_1.ZIP › Data sheet 1/Softcore_clusters_description.pdf]

T4 genes	Products
rIIA	protector from prophage-induced early lysis
39	DNA topoisomerase II large subunit
dexA	DNA exonuclease A
dda	DNA-dependent ATPase, DNA helicase
61	DNA replication primase
41	replicative DNA helicase
40	membrane-associated initiator of head vertex assembly
uvsX	RecA-like recombination protein
43	core DNA polymerase of replisome
regA	translational repressor RegA
62	clamp loader small subunit
44	DNA polymerase accessory protein
45	sliding clamp
45.2	hypothetical protein
46	endonuclease subunit
47	endonuclease subunit
a-gt.4	hypothetical protein
55	RNA polymerase sigma factor
nrdH	subunit of an anaerobic ribonucleotide reductase complex
nrdC.11	hypothetical protein
tk	thymidine kinase
tk.4	hypothetical protein
vs.1	hypothetical protein
57B	hypothetical protein
1	deoxynucleoside monophosphate kinase
3	tail sheath stabilizer, terminator
2	DNA end protector protein
4	head completion protein
53	baseplate wedge protein
5	baseplate hub subunit and tail lysozyme
6	baseplate wedge subunit
7	baseplate wedge subunit
8	baseplate wedge subunit
9	tail fiber connector and trigger for tail sheath contraction
10	baseplate wedge subunit and tail pin
11	baseplate wedge subunit and tail pin
12	short tail fibers
wac	fibritin
13	neck protein
14	neck protein
15	tail sheath stabilizer and completion protein
16	small terminase protein
17	large terminase protein
18	outer contractile sheath protein of phage tail
19	inner tube protein of phage tail
20	portal protein
68	prohead core protein
21	prohead core scaffolding protein and protease
22	head scaffolding protein

23	major capsid protein
24	capsid vertex protein
inh	inhibitor of prohead protease
uvsW	UV repair and recombination protein
uvsY	recombination, repair and ssDNA binding protein
25	baseplate wedge subunit
26	baseplate hub subunit
48	tail tube accessory protein
54	baseplate subunit
30	ATP-dependent DNA ligase
30.3	hypothetical protein
31	co-chaperone GroES
cd	deoxycytidylate deaminase
pseT	polynucleotide kinase
rnlA	RNA ligase A
nrdB	subunit of an aerobic ribonucleotide reductase complex
nrdA	subunit of an aerobic ribonucleotide reductase complex
td	I-TevI homing endonuclease
frd	dihydrofolate reductase
32	single-stranded DNA binding protein
59	helicase loading protein
33	late promoter transcription accessory protein
dsbA	double-stranded DNA binding protein
rnh	RnaseH
34	long tail fiber, proximal subunit
52	DNA topisomerase II medium subunit
rIIB	protector from prophage-induced early lysis
