# Supplementary material for: Influence of Non-canonical DNA Bases on the Genomic Diversity of Tevenvirinae
Source: Front Microbiol. 2021 Apr 6;12:632686. doi: 10.3389/fmicb.2021.632686 (PMC8056088; doi:10.3389/fmicb.2021.632686)
Supplement: Supplementary Data Sheet 4 — Characteristics of Tevenvirinae groups. [file Data_Sheet_4.docx]

**Characteristics of *Tevenvirinae* groups**

The search for homologous sequences was going on from December 14, 2019 to July 23, 2020 and, of course, during that period GenBank was supplemented with the genomes of newly sequenced *Tevenvirinae* phages. The homologous sequences of those genomes were considered as a part of GSCs. The translated ORF sequences of the genomes that were earlier removed from the analysis were also included in the GSC.

Given below is a brief description of GSCs that had homologues to the proteins of the organisms registered in the nr database. Since most of the GSCs are within groups XVII-XIX (an exception are groups represented by a single genome), we shall consider the groups in the reverse order, from XX to I. GSCs and their homologues, as well as detailed information about them, are given in Supplementary Data S3 and Supplementary Table S2.

Group XX includes only one phage, Vibrio phage vB_VmeM-32 (Akhwale et al. 2019). Basically, GSCs of this phage have homologues in the bacteriophages infecting *Vibrio* and *Vibrionaceae* bacteria, and *Caudovirales* phages isolated from marine sources. The homologous sequences of these GSCs mainly encode proteins that are presumably associated with phage activities (gene expression, morphogenesis, etc.)

Group XIX contains representatives of the genus *Schizotequatrovirus*. Apart from the phages used in clustering, other *Schizotequatrovirus* representatives: Vibrio phage nt-1 (removed at the stage of clustering; Comeau et al., 2014), Vibrio phage VH1_2019 (MN794232.1), Vibrio phage 1.081.O._10N .286.52.C2 (MG592456.1; Kauffman et al., 2018) – were found to have GSC homologues. The closest GSC homologues were mainly observed in the phages infecting bacteria isolated from marine sources. It should be noted that GSCs of this group contained a set of proteins presumably involved in the biosynthesis of 7-deazaguanine modifications (Hutinet et al., 2019). There were also homologues to the proteins participating in the biosynthesis of NAD+ and nucleotides (Skliros et al, 2016), in the morphogenesis of phage particles and in other processes.

Group XVIII contains phages that infect *Aeromonas*: Aeromonas phage Ah1 (MG250483.1), Aeromonas phage phiAS5 (NC_014636.1; Kim et al., 2012; removed at the initial stages of clustering) and Aeromonas phage AsFcp_4 (MH791407.1; Chen et al., 2018). The closest GSC homologues were mainly found in *Caudovirales* phages infecting *Gammaproteobacteria* and bacteria from marine metagenomes and human microflora. The GSCs of this group were homologous to such putative proteins as ADP ribosyltransferase, ADP-ribosylglycohydrolase, CreA protein, heme utilization protein, N-6 DNA methylase, UDP-3-O-(3-hydroxymyristoyl) glucosamine N-acyltransferase, and others.

Group XVII is also represented by phages infecting *Aeromonas*. In addition to the phages used in the procedure of clustering, this group contains Aeromonas phage AS-yj (Nolan et al., 2006), Aeromonas phage AS-sw (Chen et al., 2018) and Aeromonas phage Assk. Homologues of GCSs of this group were mainly found in the genomes of various phages infecting *Aeromonas* and bacteria from marine sources and soils. The GSCs had homologues among such putative proteins as hinge connector of long tail fiber distal connector, host protease inhibitor, adenylate cyclase, and RNase H superfamily protein.

Group XVI includes phages that infect *Enterobacteriales*; one of the representatives of this group is Enterobacteria phage RB43 (Nolan et al., 2006). Other phages: Klebsiella phage KP15 (NC_014036.1) (Maciejewska et al., 2017), KP27 (NC_020080.1), PMBT1 (NC_042138.1) (Koberg et al., 2017), KOX8 (MN101221.1), KOX10 (MN101223. 1), Yersinia phage JC221 (MN508817.1) (Yuan et al., 2020), Escherichia phage phT4A (KX130727.1) (Pereira et al., 2017), Panteoa phage Phynn (MN038175.1) – also contained GSCs of this group. Homologues of these GSCs were found in the phages of the genus *Caudovirales* infecting *Aeromonas*, *Bacillus*, *Enterobacteriales*, *Clostridioides difficile* and bacteria isolated from feces, sewage and soil – as well as *Bacillales* and *Enterobacteriales* from unknown sources. The clusters contained homologues of such proteins as DNA polymerase, radical SAM protein, DNA-cytosine methyltransferase, and base plate hub.

Group XV contains the genus *Krischvirus*. In addition to the phages used for clustering, the group includes Escherichia phage kvi (MN850615.1), Escherichia phage kaaroe (MN850574.1), Escherichia phage vB_EcoM_PHB13 (MK573636.1), Shigella phage JK32 (MK962753, Kaczorowska et al., 2019), Escherichia phage E26 (MN655998.1), Escherichia virus Ec_Makalu_001 (MN894885.1), Escherichia virus Ec_Makalu_002 (MN709127.1), and Escherichia virus Ec_Makalu_003 (MN882349.1). The closest homologues of these GSCs were among the proteins of *Myoviridae* phages (mainly *Tunavirinae* and *Vequintavirinae*) infecting *Enterobacteriales* (mainly *Salmonella enterica*, isolated from various sources).

Group XIV includes phages infecting *Proteus* and *Morganella*: Proteus phage PM2 (MF001355.1), Proteus phage phiP4-3 (MG696114.1) and Shigella phage vB_SdyM_006 (MK295204.1). Most of the homologues were found in *Caudovirales* viruses infecting *Enterobacteriales*, *Aeromonas*, *Stenothrophomonas*, *Vibrio*, and *Cellulomonas oligotrophica*. GSCs of this group were found to contain homologues of nucleoside triphosphate pyrophosphohydrolase, RIIB, ADP-ribose-binding protein, and HNH endonuclease.

Group XIII includes phages infecting *Acinetobacter*: Acinetobacter phage KARL-1 (MH713599.1) (Jansen et al., 2018), Acinetobacter phage vB_AbaP_Stupor (MN662249.1), Acinetobacter phage vB_AbaM_PhT2 (MN864865.1), Acinetobacter phage AbTZA1 (MK278860.1, Nir-Paz et al., 2019), Acinetobacter phage vB_AbaP_Kimel (MN732883.1), Acinetobacter phage vB_AbaP_Lazarus (MN782535.1), Acinetobacter phage vB_AbaP_Berthold (MN709128.1), Acinetobacter phage vB_AbaP_Konradin (MN648195.1), Acinetobacter phage vB_AbaP_Apostate (MN723850.1). The closest homologues of GSCs of this group were found among the proteins of phages isolated from *Acinetobacter* and marine sources. One of the homologues was identified as ADP-ribose pyrophosphatase.

Group XII contains a single genome, Serratia phage PS2 (Teng et al., 2018). Additionally, this group includes Serratia phage Muldoon (MN095771.1) (Campbell et al., 2020). The closest homologues were mainly found among proteins of *Myoviridae* infecting enterobacteria and proteins of enterobacteria themselves: HNH endonuclease, long tail fiber distal subunit, distal long tail fiber assembly catalyst, t holin lysis mediator.

Group XI also consists of a single genome, Pseudomonas phage PspYzu05. Most GSCs of this group had their closest homologues among the translated ORFs of metagenomes isolated from marine sources, as well as *Tevenvirinae* (the similarity was below the level established during clustering). There were also some homologues of the proteins of *Caudovirales* phages, infecting *Vibrio*, *Aeromonas* and *Enterobacteriales*, and bacteria of human microflora. The homologues included a number of predicted proteins: nucleoside triphosphate pyrophosphohydrolase, LtrC-like protein, tail fiber protein, and ABC transporter ATPase.

Group X is a single-genome group as well. It contains genome of the *Erwinia* phage *Cronus*. The closest homologues were mainly found in the phages of *Caudovirales* isolated from enterobacteria (in case of several GSCs, from *Erwinia*) and in enterobacteria themselves. The homologues included EPS (extracellular polysaccharide)-depolymerase, single-stranded DNA-binding protein, and inhibitor of host Lon protease.

Group IX contains *Jiaodavirus* phages. In addition to the phages used in clustering, this group includes Klebsiella phage JD18 (NC_028686.1), Klebsiella phage KPN5 (MN101229.1) (Kumari et al., 2010), Klebsiella phage KP179 (MH729874.1), Klebsiella phage KPN6 (MN101230.1), Klebsiella phage JIPh_Kp122 (MN434095.1) (Venturini et al., 2020), Klebsiella phage KPN2 Hku-2019 (MN101226.1). Most of the clusters had homologues among *Klebsiella* proteins; several clusters, among proteins of *Tevenvirinae*, *Winklervirus* and *Kolesnikvirus* infecting enterobacteria.

Group VIII represents *Karamvirus* phages. In addition to the phages used for clustering, this group includes Enterobacter phage prasa_myo (MN617835.1) and Klebsiella phage vB_KpnM_KaAlpha (MN013084.1). The closest GSC homologues were mostly found in *Winklervirus* phages infecting enterobacteria. Among the homologues were Head outer capsid proteins.

Group VII contains *Moonvirus* phages, and additionally includes Citrobacter phage CF1 ERZ-2017 (NC_042067.1). The closest homologues were found among *Tevenvirinae*, *Vequintavirinae* and *Tunavirinae* phages isolated from enterobacteria, and among *Dickeya* bacteria.

Group VI consists of *Gelderlandvirus* phages. Only one cluster of these group was found to have homologues among the putative proteins of *Tegunavirus*, *Winklervirus* and *Sugarlandvirus* infecting enterobacteria.

Group V contains a single genome, Pectobacterium bacteriophage PM2 (Lim et al., 2015). The closest homologues were found among the proteins of *Tevenvirinae* and *Winklervirus* phages isolated from enterobacteria. The GSCs had homologues to the predicted molybdopterin-guanine dinucleotide biosynthesis protein MobD and head completion protein.

Group IV consists of two phages infecting *Klebsiella*, and it also includes Klebsiella phage vB_KpnM_Potts1 (MN013081.1), Klebsiella phage EI (MN106245.1), Klebsiella phage PhiKpNIH-6 (MN395284.1) (2020), et al. Klebsiella phage vB_Kpn_P545 (MN781108.1), Klebsiella phage AmPh_EK29 (MN434092.1) (Venturini et al., 2020). The closest homologues of GSCs were found in the enterobacteria-infecting *Tevenvirinae* phages, and phages of *Winklervirus* infecting enterobacteria, *Yersinia* and *Clostridioides difficile*. The homologues included I-spanin.

Group III contains two genera of *Tevenvirinae* (*Gaprivervirus* and *Dhakavirus*). They had one common cluster, which was found to have homologues with the hypothetical Salmonella phage SHP1 protein (*Dhakavirus*).

Group II represents *Mosigvirus*. In addition to the phages used in clustering, this group also includes Escherichia phage phiE142 (KU255730.1) (Amarillas et al., 2016), Shigella phage Shf125875 (NC_025437.1) (Schofield et al., 2015), Escherichia phage vB_EcoM_NBG1 (MH243438.1) (Costa et al., 2018), Escherichia phage mobillu (MN850622.1), Shigella phage phi25-307 (MG589383.1). The GSCs had homologues to hypothetical proteins of *Salmonella enterica* subsp. *enterica* isolated from humans.

Group I represents *Tequatrovirus*. There were two GSCs that had homologues among the representatives of *Tequatrovirus* and bacteria *Clostridioides* *difficile* and *Salmonella enterica* that were not used in clustering (one had homologues with *Bacillus cereus*). One of the clusters had homologues to thioredoxin.

**References**

Akhwale, J. K., Rohde,M., Rohde, C., Bunk, B., Spröer, C., Boga, H. I., et al. (2019). Isolation, characterization and analysis of bacteriophages from the haloalkaline lake Elmenteita, Kenya. PLoS One 14:e0215734.

Amarillas, L., Chaidez, C., González-Robles, A., and León-Félix, J. (2016). Complete genome sequence of new bacteriophage phiE142, which causes simultaneously lysis of multidrug-resistant Escherichia coli O157:H7 and Salmonella enterica. Stand. Genom. Sci. 11:89. doi: 10.1186/s40793-016-0211-5

Campbell, S., Atkison, C., Moreland, R., Liu, M., Ramsey, J., and Leavitt, J. (2020). Complete genome sequence of Serratia phage muldoon. Microbiol. Resour. Announc. 9:e01418-19. doi: 10.1128/MRA.01418-19

Chen, L., Yuan, S., Liu, Q., Mai, G., Yang, J., Deng, D., et al. (2018). In vitro design and evaluation of phage cocktails against Aeromonas salmonicida. Front. Microbiol. 9:1476. doi: 10.3389/fmicb.2018.01476

Comeau, A. M., Arbiol, C., and Krisch, H. M. (2014). Composite conserved promoter-terminator motifs (PeSLs) that mediate modular shuffling in the diverse T4-like myoviruses. Genome biology and evolution, 6(7), 1611–1619. doi:10.1093/gbe/evu129

Costa, A. R., Brouns, S. J. J., and Nobrega, F. L. (2018). Complete genome sequences of two T4-Like Escherichia coli bacteriophages. Genome Announc. 6:e0586-18. doi: 10.1128/genomeA.00586-18

Hutinet, G., Kot, W., Cui, L., Hillebrand, R., Balamkundu, S., Gnanakalai, S., et al. (2019). 7-Deazaguanine modifications protect phage DNA from host restriction systems. Nat. Commun. 10:5442. doi: 10.1038/s41467-019-13384-y

Jansen, M., Wahida, A., Latz, S., Krüttgen, A., Häfner, H., Buhl, E. M., et al. (2018). Enhanced antibacterial effect of the novel T4-like bacteriophage KARL-1 in combination with antibiotics against multi-drug resistant Acinetobacter baumannii. Sci. Rep. 8:14140. doi: 10.1038/s41598-018-32344-y

Kaczorowska, J., Casey, E., Neve, H., Franz, C., Noben, J. P., Lugli, G. A., et al. (2019). A quest of great importance-developing a broad spectrum Escherichia coli phage collection. Viruses 11:899. doi: 10.3390/v11100899

Kauffman, K. M., Hussain, F. A., Yang, J., Arevalo, P., Brown, J. M., Chang, W. K., et al. (2018). A major lineage of non-tailed dsDNA viruses as unrecognized killers of marine bacteria. Nature 554, 118–122. doi: 10.1038/nature25474

Kim, J. H., Son, J. S., Choi, Y. J., Choresca, C. H. Jr., Shin, S. P., Han, J. E., et al. (2012). Complete genome sequence and characterization of a broad-host range T4-like bacteriophage phiAS5 infecting Aeromonas salmonicida subsp. salmonicida. Vet. Microbiol. 157, 164–171. doi: 10.1016/j.vetmic.2011.12.016

Koberg, S., Brinks, E., Fiedler, G., Hüsing, C., Cho, G. S., Hoeppner, M. P., et al. (2017). Genome sequence of Klebsiella pneumoniae bacteriophage PMBT1 isolated from raw sewage. Genome Announc. 5:e0914-16. doi: 10.1128/genomeA.00914-16

Kumari, S., Harjai, K., and Chhibber, S. (2010). Isolation and characterization of Klebsiella pneumoniae specific bacteriophages from sewage samples. Folia Microbiol. 55, 221–227. doi: 10.1007/s12223-010-0032-7

Lim, J. A., Lee, D. H., and Heu, S. (2015). Isolation and genomic characterization of the T4-Like bacteriophage PM2 infecting Pectobacterium carotovorum subsp. carotovorum. Plant Pathol. J. 31, 83–89. doi: 10.5423/PPJ.NT.09.2014.0099

Maciejewska, B., Roszniowski, B., Espaillat, A., Kêsik-Szeloch, A., Majkowska-Skrobek, G., Kropinski, A. M., et al. (2017). Klebsiella phages representing a novel clade of viruses with an unknown DNA modification and biotechnologically interesting enzymes. Appl. Microbiol. Biotechnol. 101, 673–684. doi: 10.1007/s00253-016-7928-3

Nir-Paz, R., Gelman, D., Khouri, A., Sisson, B. M., Fackler, J., Alkalay-Oren, S., et al. (2019). Successful treatment of antibiotic-resistant, poly-microbial bone infection with bacteriophages and antibiotics combination. Clin. Infect. Dis. 69, 2015–2018. doi: 10.1093/cid/ciz222

Nolan, J. M., Petrov, V., Bertrand, C., Krisch, H. M., and Karam, J. D. (2006). Genetic diversity among five T4-like bacteriophages. Virol. J. 3:30. doi: 10.1186/1743-422X-3-30

Pereira, C., Moreirinha, C., Lewicka, M., Almeida, P., Clemente, C., Romalde, J. L., et al. (2017). Characterization and in vitro evaluation of new bacteriophages for the biocontrol of Escherichia coli. Virus Res. 227, 171–182. doi: 10.1016/j.virusres.2016.09.019

Schofield, D. A., Wray, D. J., and Molineux, I. J. (2015). Isolation and development of bioluminescent reporter phages for bacterial dysentery. Eur. J. Clin. Microbiol. Infect. Dis. 34, 395–403. doi: 10.1007/s10096-014-2246-0

Skliros, D., Kalatzis, P. G., Katharios, P., and Flemetakis, E. (2016). Comparative functional genomic analysis of two Vibrio phages reveals complex metabolic interactions with the host cell. Front. Microbiol. 7:1807. doi: 10.3389/fmicb.2016.01807

Teng, T., Zhang, G., Fan, X., Zhang, Z., Zhang, L., Wu, D., et al. (2018). Complete genome sequence analysis of PS2, a novel T4-like bacteriophage that infects Serratia marcescens clinical isolates. Arch. Virol. 163, 1997–2000. doi: 10.1007/s00705-018-3803-0

Venturini, C., Ben Zakour, N. L., Bowring, B., Morales, S., Cole, R., Kovach, Z., et al. (2020). Fine capsule variation affects bacteriophage susceptibility in Klebsiella pneumoniae ST258. FASEB J. 34, 10801–10817. doi: 10.1096/fj.201902735R

Yuan, Y., Xi, H.,Dai, J., Zhong, Y., Lu, S., Wang, T., et al. (2020). The characteristics and genome analysis of the novel Y. pestis phage JC221. Virus Res. 283:197982. doi: 10.1016/j.virusres.2020.197982
