# Supplementary material for: Influence of Non-canonical DNA Bases on the Genomic Diversity of Tevenvirinae
Source: Front Microbiol. 2021 Apr 6;12:632686. doi: 10.3389/fmicb.2021.632686 (PMC8056088; doi:10.3389/fmicb.2021.632686)
Supplement: Supplementary Data Sheet 5 — Characteristics of the region of genes of the synthesis of hmdC and its modifications of Tevenvirinae. [file Data_Sheet_5.DOCX]

Characteristic of region of modified ^hm^dC synthesis genes

The studies of genes associated with the synthesis of base modifications revealed their bacterial origin, location in certain genomic islands, and the possibility of their horizontal transfer (Thiaville et al., 2016). By analogy, we assume that the genes of proteins involved in the synthesis of modified ^hm^dC have similar characteristics. These characteristics are more difficult to identify, however – since the phages carrying those genes underwent large changes in their activities, as compared to their ancestors.

As we found, most of the genes encoding homologues of proteins associated with the synthesis of ^hm^dCMP and its modifications (with the exception of genes of α-glucosyl transferase and, in some cases, arabinosyl transferase) were located in the region between the core gene encoding helicase and a module of core genes that included the gene of DNA polymerase. More precisely, these genes were mainly located between the genes of DNA polymerase and UvsX (or helicase, in case of UvsX absence). Two representatives of group XIII (Acinetobacter phages AM101 and Acj61), contained a couple of homologous arabinosyl transferase genes in this region. Considering the place of these phages on the pan-genomic tree, one can suppose that the location of arabinosyl transferase gene outside the considered part of the genome is associated with its transfer by homing endonucleases. The presence of genes encoding these enzymes in the region may be an indication of the transfer. In addition, we found insertions of these genes in the ORF of the uvsX gene of Proteus phage vB_PmiM_Pm5461. As shown earlier, homing endonucleases were involved in the formation of a double-cistronic DNA polymerase gene in some *Tevenvirinae* representatives (Petrov et al., 2006; Petrov et al., 2010). The insertion and then self-excision from the genome could lead to a shift of ORF in some genomes. In Escherichia phage vB_EcoM_G2540, for example, the β-gt gene was split into two ORFs during annotation. We also found that the considered region contained the *imm* gene, the product of which is involved in the suppression of superinfection and in phage immunity (Cornett, 1974; Abedon, 2019). As shown by Comeau et al., 2014, a homologue of this gene in Yersinia PST phage contains the PeSL element which, presumably, enables horizontal gene transfer by recombination. Considering all the aforesaid, one can suppose that horizontal gene transfer is actively going on in this region of the genome. In *Tevenvirinae* phages, however, this transfer is rather limited – due to several circumstances.

First, as mentioned above, bacteriophages with different DNA base composition have a barrier hindering gene transfer. Second, the probability of HGT between phages of the same population is low – due to impossibility of superinfection, which is suppressed by the Sp and Imm proteins (Lu and Henning, 1994). Third, there is a possibility that closely related phages are excluded from infection by an unknown mechanism. An assumption was made that this mechanism may be related to the presence of IPI (Thomas et al., 2018). As a result, we see the emergence of a network of genetic information exchange between the phages that are not closely related but have similar sets of non-canonical bases and share the same ecological niche. The restriction of gene transfer is important for the evolution of phages: because of a faster divergence between closely related viruses, they may acquire evolutionary advantages which will not be extended to all phages of a niche. Hence, to study the possibilities of HGT in this genomic region and in the genomes of *Tevenvirinae* with ^hm^dC, it is necessary to consider metagenomes from the same ecological niche – or, alternatively, a pan-genomic analysis would be required. Another option is to study multi-infection by distantly related phages which have the same set of non-canonical bases and infect the same host bacteria.

In phages with other non-canonical bases, the synthesis of which is mediated by the representatives of the same thymidylate synthase family as dCMP hydroxymethylase (Lee et al., 2018), the genes of synthases are located near the genes of DNA polymerase or Uvsx. Located next to them are the genes of thymidylate kinases, which are also involved in the synthesis of thymidine hypermodifications. As we found, the region considered does contain a hypothetical thymidylate kinase. This may indicate that the clustering of genes, which are associated with the synthesis of non-canonical bases via the thymidylate-synthase pathway, into a separate genomic island is quite an ancient event.

Thus, the fragment of the genome that is associated with the synthesis of ^hm^dC and its modifications (a region between the gene encoding homologues of helicase and the module of core sequences that includes the gene of DNA polymerase) may be a genomic island of ancient origin, whose initial functions are difficult to determine. Genes of this region are subjected to horizontal transfer, but its detection is difficult due to the nature of the viruses that have this region. Taking into account the two circumstances: (1) the clustering of genes, whose products are involved in the synthesis of ^hm^dC and its modifications, in the same region and (2) the bacterial origin of phage genomic islands, containing genes associated with the synthesis of 7-deazaguanine modifications – one can assume a similar mechanism of emergence of ^hm^dC in *Tevenvirinae*.

**References**

Abedon, S. T. (2019). Look Who's Talking: T-Even Phage Lysis Inhibition, the Granddaddy of Virus-Virus Intercellular Communication Research. Viruses 11:951. doi:10.3390/v11100951

Comeau, A. M., Arbiol, C., and Krisch, H. M. (2014). Composite conserved promoter-terminator motifs (PeSLs) that mediate modular shuffling in the diverse T4-like myoviruses. Genome Biol Evol. 6, 1611-1619. doi: 10.1093/gbe/evu129.

Cornett, J. B. (1974). Spackle and immunity functions of bacteriophage T4. J Virol. 13, 312-321. doi:10.1128/JVI.13.2.312-321.1974

Lee, Y. J., Dai, N., Walsh, S. E., Müller, S., Fraser, M. E., Kauffman, K. M., Guan C., Corrêa, I. R. Jr., and Weigele, P. R. (2018). Identification and biosynthesis of thymidine hypermodifications in the genomic DNA of widespread bacterial viruses. Proc Natl Acad Sci U S A 115, E3116-E3125. doi:10.1073/pnas.1714812115

Lu, M. J., and Henning, U. (1994). Superinfection exclusion by T-even-type coliphages. Trends Microbiol. 2, 137-139. doi:10.1016/0966-842x(94)90601-7

Petrov, V. M., Ratnayaka, S., and Karam, J. D. (2010). Genetic insertions and diversification of the PolB-type DNA polymerase (gp43) of T4-related phages. J Mol Biol. 395, 457-474. doi:10.1016/j.jmb.2009.10.054

Thiaville, J. J., Kellner, S. M., Yuan, Y., et al. (2016). Novel genomic island modifies DNA with 7-deazaguanine derivatives. Proc Natl Acad Sci U S A 113, E1452-E1459. doi:10.1073/pnas.1518570113

Thomas, J. A., Orwenyo, J., Wang, L.X., and Black, L.W. (2018). The Odd "RB" Phage-Identification of Arabinosylation as a New Epigenetic Modification of DNA in T4-Like Phage RB69. Viruses 10:313. doi:10.3390/v10060313
