# Supplementary material for: Influence of Non-canonical DNA Bases on the Genomic Diversity of Tevenvirinae
Source: Front Microbiol. 2021 Apr 6;12:632686. doi: 10.3389/fmicb.2021.632686 (PMC8056088; doi:10.3389/fmicb.2021.632686)

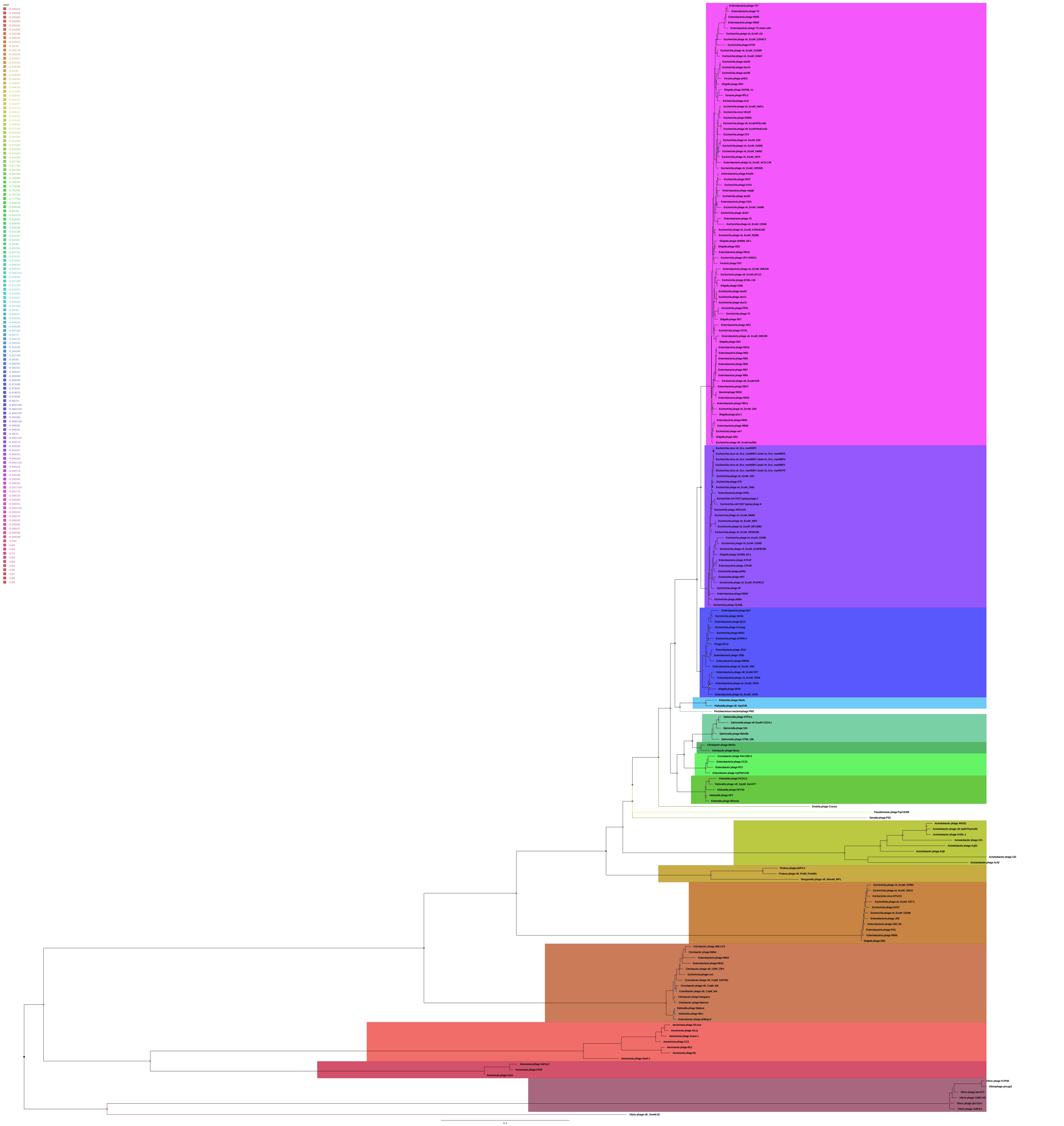

Supplement: Supplementary Figure 2 — The pan-genomic tree of Tevenvirinae (shown in Figure 1) with labeled aBayes support/ultrafast bootstrap support. Black circles indicate the root and the nodes of branches with the same sequences as leaves. [file Image_2.PDF]
