# Supplementary material for: Influence of Non-canonical DNA Bases on the Genomic Diversity of Tevenvirinae
Source: Front Microbiol. 2021 Apr 6;12:632686. doi: 10.3389/fmicb.2021.632686 (PMC8056088; doi:10.3389/fmicb.2021.632686)
Supplement: Supplementary Figure 3 — The phylogenetic tree of Tevenvirinae (presented in Figure 2) with labeled SH-aLRT support/aBayes support/ultrafast bootstrap support. Black circles indicate the root and the nodes of branches with the same sequences as leaves. [file Image_3.PDF]

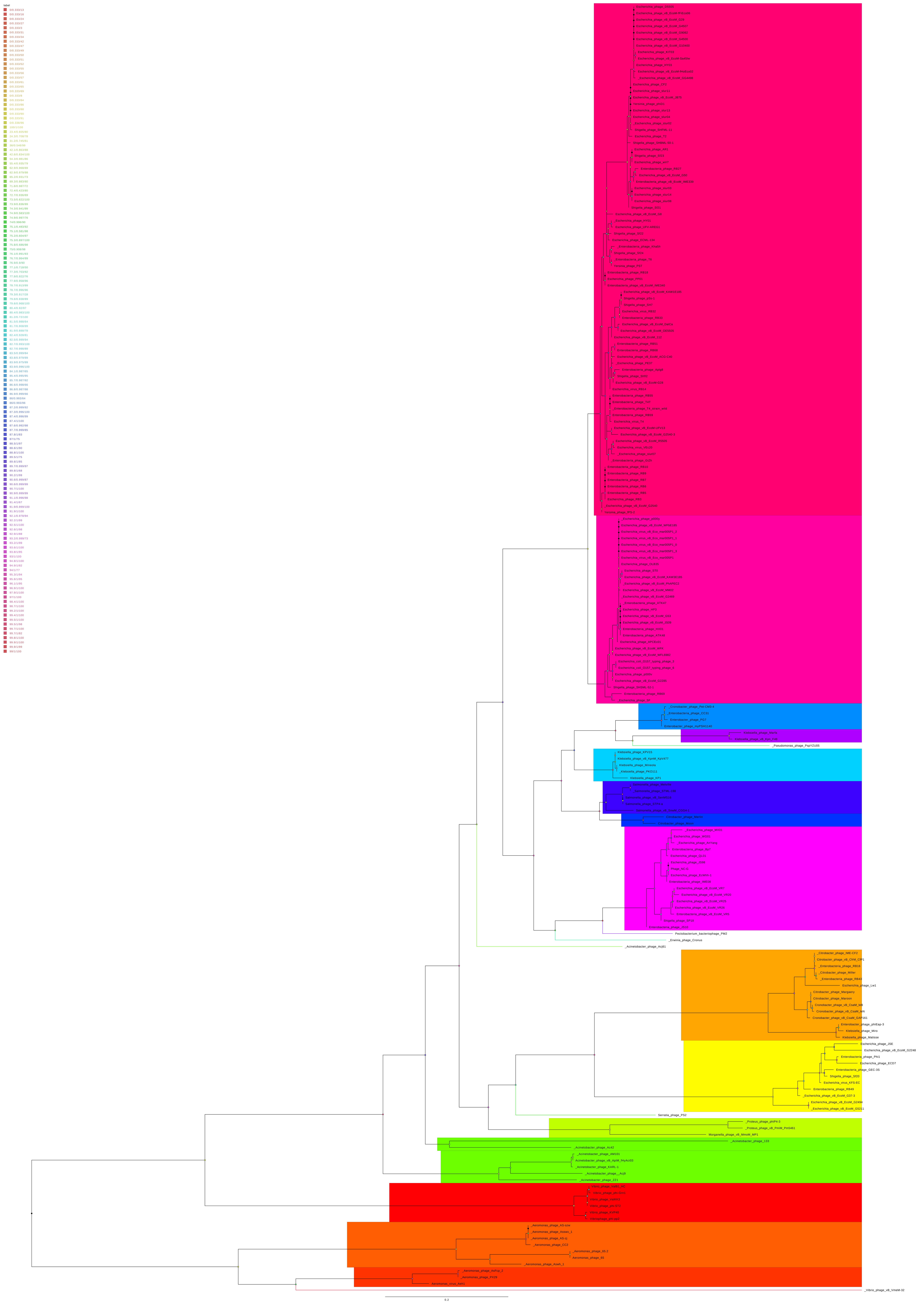

label
0.2
_Vibrio_phage_vB_VmeM-32
Aeromonas_virus_Aeh1
Serratia_phage_PS2
Pectobacterium_bacteriophage_PM2
_Erwinia_phage_Cronus
_Acinetobacter_phage_Acj61
_Pseudomonas_phage_PspYZU05
